# *Kingella kingae* RtxA Cytotoxin in the Context of Other RTX Toxins

**DOI:** 10.3390/microorganisms10030518

**Published:** 2022-02-27

**Authors:** Katerina Filipi, Waheed Ur Rahman, Adriana Osickova, Radim Osicka

**Affiliations:** Institute of Microbiology of the Czech Academy of Sciences, Videnska 1083, 142 20 Prague, Czech Republic; katerina.filipi@biomed.cas.cz (K.F.); waheed.rahman@biomed.cas.cz (W.U.R.); osickova@biomed.cas.cz (A.O.)

**Keywords:** β_2_ integrins, *Kingella kingae*, membrane, pore-forming, RtxA, RTX toxin

## Abstract

The Gram-negative bacterium *Kingella kingae* is part of the commensal oropharyngeal flora of young children. As detection methods have improved, *K. kingae* has been increasingly recognized as an emerging invasive pathogen that frequently causes skeletal system infections, bacteremia, and severe forms of infective endocarditis. *K. kingae* secretes an RtxA cytotoxin, which is involved in the development of clinical infection and belongs to an ever-growing family of cytolytic RTX (Repeats in ToXin) toxins secreted by Gram-negative pathogens. All RTX cytolysins share several characteristic structural features: (i) a hydrophobic pore-forming domain in the N-terminal part of the molecule; (ii) an acylated segment where the activation of the inactive protoxin to the toxin occurs by a co-expressed toxin-activating acyltransferase; (iii) a typical calcium-binding RTX domain in the C-terminal portion of the molecule with the characteristic glycine- and aspartate-rich nonapeptide repeats; and (iv) a C-proximal secretion signal recognized by the type I secretion system. RTX toxins, including RtxA from *K. kingae*, have been shown to act as highly efficient ‘contact weapons’ that penetrate and permeabilize host cell membranes and thus contribute to the pathogenesis of bacterial infections. RtxA was discovered relatively recently and the knowledge of its biological role remains limited. This review describes the structure and function of RtxA in the context of the most studied RTX toxins, the knowledge of which may contribute to a better understanding of the action of RtxA in the pathogenesis of *K. kingae* infections.

## 1. Introduction

The fastidious and facultatively anaerobic Gram-negative coccobacillus *Kingella kingae*, from the family of *Neisseriaceae*, was first isolated by Elizabeth King in 1960 [1,2,3]. In 1968, Bovre and Henriksen classified this bacterium as a member of the genus *Moraxella* and named it *Moraxella kingii* in honor of Elizabeth King [2]. To avoid confusion with *Pseudomonas kingii*, the name of the bacterium was changed to *Moraxella kingae* in 1974, and the bacterium was finally assigned to the genus *Kingella* in 1976 [4,5]. *K. kingae* is part of the commensal oropharyngeal flora of young children, and the bacterium was initially thought to only rarely cause systemic infections [3,6,7]. Nevertheless, advances in culture techniques and molecular detection methods revealed that *K. kingae* is a common cause of septic arthritis and osteomyelitis in children [3,7,8,9,10,11,12,13]. The bacterium also accounts for other invasive diseases such as infective endocarditis, bacteremia, pneumonia, meningitis, pericarditis, peritonitis, or ocular infections [1,11]. The transmission of *K. kingae* occurs through close personal contact, and the highest colonization occurs between 6 and 36 months of age [1,3,14,15]. The carriage steadily decreases in older children and adults, probably due to the acquisition of immunity that eliminates the bacterium from the pharynx [14,16].

Based on the observation that genotypically identical pharyngeal and bloodstream isolates were found in three children with invasive *K. kingae* infections, invasive disease is thought to begin with asymptomatic colonization of the upper respiratory tract [17]. The process of colonization involves adherence of *K. kingae* to the host airway epithelium via type IV pili and a trimeric autotransporter adhesin called Knh [18,19]. After colonization, *K. kingae* breaches the respiratory epithelial barrier by an unknown mechanism to invade the underlying lamina propria. It remains unclear whether the bacteria can directly penetrate into the underneath blood capillaries or reach the blood circulation through the draining lymphatics. Several previous reports have shown that patients with invasive *K. kingae* disease also had symptoms of viral respiratory infection, herpetic gingivostomatitis, or concomitant buccal aphthous ulcers [20,21]. Therefore, it was hypothesized that viral-induced damage to the respiratory mucosa might facilitate tissue penetration and entry of *K. kingae* into the bloodstream [1]. Once there, *K. kingae* may cause bacteremia, or it is disseminated to distant sites in the body, such as bones, joints, or the endocardium [8,9,10,22].

**Figure 1:**
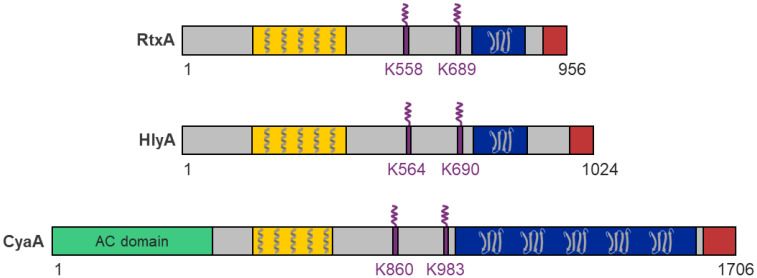
The structural representation of the RTX toxins RtxA, HlyA, and CyaA. The RtxA, HlyA, and CyaA toxins consist of a pore-forming domain (yellow), an acylated segment with two posttranslationally acylated lysine residues (purple), an RTX domain (blue), and a C-terminal secretion signal (red). Unlike other RTX toxins, CyaA contains a unique adenylate cyclase domain (green).

Microscopic observations and lactic acid dehydrogenase release assays showed that *K. kingae* is cytotoxic to cultured synovial, macrophage, and respiratory epithelial cells, and the cytotoxic effect was attributed to the RTX (Repeats in ToXin) cytotoxin RtxA (Figure 1) [23]. Using mariner-based transposon mutagenesis, the *rtx* locus encoding RtxA was first identified in the *K. kingae* strain 269–492 [23]. Later, the *rtx* locus was detected in all tested clinical isolates of *K. kingae*, and recently, it was also identified in a new species named *K. negevensis* [24,25,26,27]. Later experiments using the infant rat model and the RtxA-deficient mutant KKNB100 showed that the RtxA cytotoxin is a key virulence factor of *K. kingae* [28]. It was hypothesized that the toxin might facilitate the disruption of the respiratory epithelium to allow *K. kingae* to invade the bloodstream [23].

RtxA belongs to a broad family of pore-forming RTX cytotoxins secreted by many Gram-negative pathogens, including the bacteria of the genera *Actinobacillus*, *Aggregatibacter*, *Bordetella*, *Escherichia*, *Mannheimia*, *Moraxella*, *Morganella*, *Pasteurella*, *Proteus*, and *Vibrio* [29,30]. For all RTX toxins, several functional domains and characteristic segments can be defined (Figure 1) [29,30]:

1. A hydrophobic pore-forming domain in the N-terminal part of the molecule that harbors several putative transmembrane α-helices;
2. An acylated segment where the RTX protoxin is activated and converted into the RTX toxin by a co-expressed toxin-activating acyltransferase that catalyzes the covalent posttranslational acylation of conserved lysine residues;
3. A typical C-terminal calcium-binding RTX domain containing various numbers of the conserved glycine- and aspartate-rich nonapeptide repeats of a consensus sequence G-G-X-G-X-D-X-U-X (X represents any residue and U represents the hydrophobic residue leucine, valine or isoleucine), which form calcium-binding sites;
4. A C-proximal unprocessed secretion signal for export of the RTX toxin from the bacterial cell by the type I secretion system (T1SS).

Upon binding to host cells, RTX cytolysins insert into the cell membrane and form cation-selective pores that trigger cation fluxes (calcium influx and potassium efflux) across the cell membrane that disrupt normal cell physiology and ultimately cause cell death [29,30,31,32,33,34,35,36,37]. Based on species and cellular specificity, RTX toxins can be roughly divided into two groups: (i) RTX hemolysins, which are capable of lysing erythrocytes and exhibit toxicity to various cell types isolated from different species, and (ii) RTX leukotoxins, which exhibit narrow species and cell specificity because they bind via leukocyte-restricted β_2_ integrins [29,30,38].

In this review, we discuss structural-functional aspects of RtxA from *K. kingae* in the context of the best-studied RTX hemolysins and leukotoxins that are listed in Table 1.

**Table 1:** General characteristics of RTX toxins discussed in the text.

RTX Toxin	Bacterium; Disease	Size (kDa)	Acylated Residues	Species and Cell Specificity ^1^	Ref.
**RtxA**	***Kingella kingae***; Osteoarticular infections, endocarditis and others	105	K558 K689	**Broad**: human epithelial and monocyte cell lines, mouse monocyte/macrophage cell line, rabbit fibroblast cell line, sheep erythrocytes	[1,23,36,37,39]
**HlyA**	**Uropathogenic** ***Escherichia coli***; Urinary tract infections	110	K564 K690	**Broad**: primary human epithelial cells and leukocytes, primary rat epithelial cells, primary porcine endothelial cells, human epithelial, promonocytic myeloid, T- and B-lymphocyte cell lines, porcine endothelial cell line, erythrocytes of various species	[40,41,42,43,44,45,46,47,48]
**CyaA**	***Bordetella pertussis***; Whooping cough	177	K860 K983	**Narrow**: primary human myeloid cells, human monocyte and splenic myeloid dendritic cell lines, mouse macrophage cell line	[49,50,51,52,53,54,55,56,57]
**LtxA**	***Aggregatibacter*** ***actinomycetemcomitans***; Aggressive periodontitis	116	K562 K687	**Narrow**: primary human monocytes, primary human and primate polymorphonuclear leukocytes, human T- and B-lymphocyte, monocyte, and promyeloblast cell lines	[58,59,60,61,62,63,64,65,66]
**LktA**	***Mannheimia haemolytica***; Pneumonic pasteurellosis	102	K554 K669	**Narrow**: primary ruminant leukocytes and platelets, bovine B-lymphosarcoma cell line	[67,68,69,70,71]
**ApxIA**	***Actinobacillus*** ***pleuropneumoniae***; Porcine pleuropneumonia	110	K560 K686	**Broad**: primary porcine alveolar macrophages and neutrophils, primary bovine and porcine endothelial cells, sheep, swine and horse erythrocytes	[72,73,74,75,76,77,78]
**ApxIIIA**	***Actinobacillus*** ***pleuropneumoniae***; Porcine pleuropneumonia	120	K571 K702	**Narrow**: primary porcine and wild boar peripheral blood mononuclear cells, primary bovine and porcine endothelial cells	[73,77,79,80]

^1^ RTX toxins with narrow species and cell specificity that specifically bind to β_2_ integrins on the cell surface were found to exhibit detectable binding and cytotoxic activity also on β_2_ integrin-negative cells (e.g., erythrocytes of various species), as described in the text and evident from the references in Table 1.

## 2. Genes Required for RtxA Production, Activation and Secretion

The *K. kingae rtx* locus contains five genes, namely, *rtxA*, *rtxB*, *rtxC*, *rtxD*, and *tolC*, all of which are involved in the production, activation, and secretion of the RtxA toxin [23]. The *rtxA* gene encodes an inactive form of the toxin, called protoxin (proRtxA), which is activated by the toxin-activating acyltransferase RtxC, encoded by the *rtxC* gene. The remaining three genes encode the RtxB, RtxD, and TolC proteins, which form the T1SS that transports the RtxA toxin from the cytosol directly through the bacterial envelope into the extracellular environment. The *rtx* locus of *K. kingae* is flanked by insertion elements homologous to the insertion elements of *Moraxella bovis*. Moreover, the *rtxA*, *rtxC*, and *rtxB* genes of *K. kingae* are more than 70% identical to the corresponding genes of *M. bovis*, and the *rtxD* and *tolC* genes are 64% and 81% identical, respectively, to its homologues from *Neisseria meningitis*. Therefore, it can be assumed that the *rtx* locus of *K. kingae* was acquired from a donor species by horizontal gene transfer [3,23].

**Figure 2:**
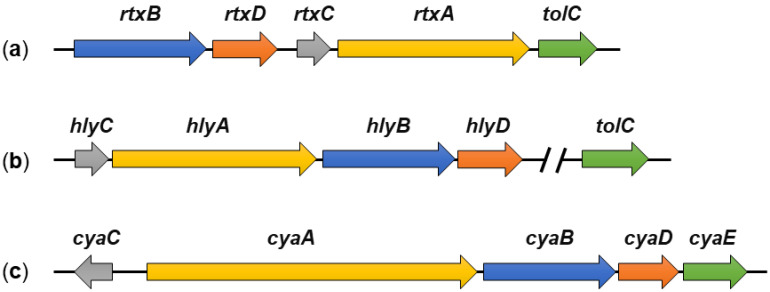
Genetic organization of the *rtx* loci. The schematic representation of the *rtx* gene locus of *K. kingae* strain 269–492 (**a**), *E. coli* strain CFT073 (**b**), and *B. pertussis* strain 18323 (**c**). The colored arrows represent coding regions and transcriptional directions of the *rtx* genes encoding the protoxin proRTXA (yellow), the acyltransferase RTXC (gray), and the proteins of the T1SS apparatus (blue, ABC transporter; orange, MFP; green, OMF).

It is noteworthy that all five *rtx* genes of *K. kingae* strain 269–492 are located in a single gene locus [23], which is different from *rtx* loci of some other RTX toxins (Figure 2). For example, the *tolC* gene is located outside the *hly* locus encoding the HlyCABD proteins of *E. coli* (Figure 2b) [29,30,81,82], or the *cyaC* gene, encoding the CyaC acyltransferase of *B. pertussis,* is oriented in the opposite direction to the *cyaABDE* genes (Figure 2c) [29,30,83].

**Figure 3:**
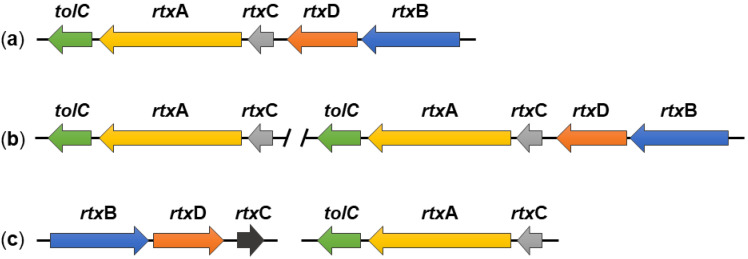
The schematic representation of the *rtx* gene loci of different *K. kingae* strains. The *rtx* loci of *K. kingae* strain 269–492 (**a**), *K. kingae* strains ATCC 23332 and KWG1 (**b**), *K. kingae* strains ATCC 23331 and NCTC 10529 (**c**). The colored arrows represent coding regions and transcriptional directions of the *rtx* genes encoding the protoxin proRtxA (yellow), the acyltransferase RtxC (light gray, a 167 residue-long variant; dark gray, a 162 residue-long variant), and the proteins of the T1SS apparatus (blue, ABC transporter; orange, MFP; green, OMF).

Nevertheless, the organization of the *rtx* loci of *K. kingae* is not fully conserved, and our in silico analysis of *K. kingae* genomes (available at the National Center for Biotechnology Information, Bethesda, Rockville, MD, USA) revealed significant differences among *K. kingae* strains (Figure 3). For example, *K. kingae* strains ATCC 23332 and KWG1 have a separate locus containing only the *rtxC*, *rtxA*, and *tolC* genes in addition to a fully preserved *rtx* locus homologous to that of *K. kingae* 269–492 (Figure 3b). Furthermore, *K. kingae* strains ATCC 23331 and NCTC 10529 do not have a contiguous *rtx* locus, and the genes encoding the RtxABCD/TolC proteins are split into two loci. The first contains the *rtxC*, *rtxA*, and *tolC* genes and the second the *rtxB*, *rtxD*, and *rtxC* genes (Figure 3c). This goes well with the previous study of Opota and colleagues who examined the genomes of *K. kingae* strains KWG1 and ATCC 23330, also referred to as NCTC 10529 [26]. All these data suggest that the *rtx* locus of *K. kingae* is plastic, and other *rtx* loci arrangements may be found in additional isolates of *K. kingae*.

**Figure 4:**
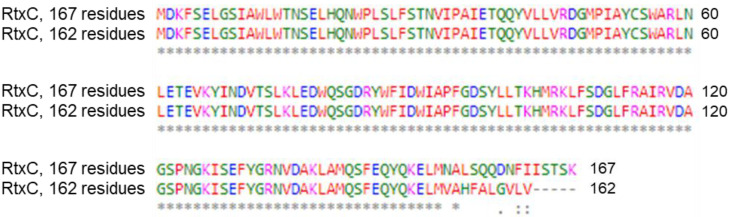
The ClustalW sequence alignment of two RtxC variants of *K. kingae* strain ATCC 23331. Symbols: (*) identity; (:) strongly similar; (.) weakly similar.

In addition, we found two different alleles of the *rtxC* gene that encode the acyltransferase RtxC. The first allele occurs in two copies in *K. kingae* strains KWG1 and ATCC 23332 and encodes a 167 residue-long RtxC polypeptide. The second allele, encoding a 162 residue-long RtxC polypeptide, occurs in parallel to the first type of the *rtxC* allele in strains ATCC 23331 and NCTC 10529. The amino acid sequence of the two forms of RtxC diverges by 15 amino acid residues in the C-terminal portion of the protein (Figure 4), and it remains unclear whether the 162 residue-long form still yields an active RtxC acyltransferase enzyme that can modify proRtxA.

## 3. Polymorphism of the *rtxA* Gene

An analysis of the *rtxA* alleles of 31 clinical isolates of *K. kingae* revealed 18 single-nucleotide polymorphisms in the 943 bp sequenced region of the *rtxA* gene (2871 bp in total), 6 of which result in amino acid substitutions in the RtxA protein [84]. This suggests that RtxA tends to evolve to evade the immune response, like other virulence factors [85,86,87]. Also of note is an insertion of 33 bp in position 67 of the *rtxA* gene, encoding a short sequence of 11 amino acid residues (RAGQAGVQALN) in the N-terminal portion of the RtxA toxin [84]. The insertion was found in a single copy in the *rtxA* gene of six clinical isolates of *K. kingae* and duplicated in two strains. A similar polymorphism of the *rtxA* gene was later described when the same 943 bp-long region of *rtxA* [84] was amplified and sequenced for 103 *K. kingae* strains of different clinical origin [88]. This revealed the presence of 18 different *rtxA* alleles, some of which had an insertion of 33 bp [88] previously observed by Lehours et al. [84]. However, Basmaci et al. suggested that the insertion occurred at position 76 of the *rtxA* gene instead of position 67 and was a duplication or triplication of the immediately preceding 33 bp fragment encoding the sequence QAG(V/A)QALN(R/K)AG [88]. It remains unclear whether this multiplication of the 11 residue-long sequence affects the cytotoxic activity of RtxA.

Another study examined the genetic diversity of invasive *K. kingae* strains isolated from Israeli patients with bacteremia, skeletal system infections, or endocarditis between 1991 and 2012 [87]. The 181 isolates were subdivided into 32 distinct clones by pulsed-field gel electrophoresis (PFGE), multilocus sequence typing (MLST), and *rtxA* gene sequencing. The isolates belonging to a particular PFGE clone shared the same MLST combinations and had identical or closely related *rtxA* alleles. The five predominant PFGE clones, namely, B, H, K, N, and P, caused 72.9% of all invasive infections. Interestingly, the K, N, and P clones were associated with specific clinical syndromes. Isolates of clone K mainly caused bacteremia, representatives of clone N caused skeletal system infections, and clone P was predominantly associated with endocarditis. In addition, all members of the clone K had a duplication or triplication of the 11 residue-long sequence previously reported by Lehours et al. and Basmaci et al. [84,87,88].

## 4. The *rtxA* Gene as a Diagnostic Marker of *K. kingae*

*K. kingae* is a fastidious microorganism, and its detection by classical culture methods has often been unsuccessful [89]. However, the development and application of new molecular methods, such as PCR, has greatly improved the identification of the bacterium [3]. The first PCR tests for the detection of *K. kingae* were based on the analysis of the *16S rRNA* gene [90,91,92] and the gene encoding the chaperone GroEL, also called Cpn60 [93,94,95,96]. Later, the *rtx* locus was found to be associated exclusively with *K. kingae* and not with other *Kingella* species, and the *rtx* genes began to serve as diagnostic markers for diseases caused by *K. kingae* [84]. Real-time PCR assays specific for the *rtxA* gene [84] or for the *rtxA* and *rtxB* genes [24,25] were gradually developed to identify clinical isolates of *K. kingae*. For example, Slinger and colleagues searched for the *rtxA* gene in 50 clinical samples, and if positive, they also examined the presence of the *rtxB* gene for confirmation [97]. Haldar et al. developed a multiplex PCR for the *rtxA* and *cpn60* genes of *K. kingae* and the *spa* gene of *Staphylococcus aureus*, which is also a common causative agent of septic arthritis in children [98,99]. These methods were effective and improved the detection rate of diseases caused by *K. kingae* [97,100].

In 2017, *Kingella negevensis* strain *eburonensis*, a new species of the genus *Kingella*, was described [27], and an analysis of its genome revealed the presence of an *rtx* locus highly homologous to that of *K. kingae* (strains ATCC 23330 and KWG1) [26]. Hence, molecular tests targeting the *rtx* locus could not distinguish between *K. kingae* and *K. negevensis* [101]. Therefore, a duplex real-time PCR was developed in which the *rtxA* and *cpn60* genes were targeted [26]. Meanwhile, 18 different variants of the malate dehydrogenase (*mdh*) gene were found in the genomes of 20 different sequence types of *K. kingae*, but not in the genomes of other *Kingella* species [102]. The specifically designed *mdh*-based primers without nucleotide mismatches were then successfully used to diagnose *K. kingae* carriage and infections in 104 clinical samples from children around the world aged between 7 months and 7 years [102]. The real-time PCR assay targeting the *mdh* gene was found to be highly specific only for *K. kingae* and not for other *Kingella* species, including *K. negevensis* [102]. This approach was recently used to describe invasive *K. kingae* infections in a daycare center in France [103]. *K. negevensis* shares more virulence factors with *K. kingae* than the RtxA toxin encoded by the *rtx* locus, for example, the capsule or type IV pili [104]. Therefore, the question remains of what is the pathogenic potential of *K. negevensis* and how does it differ from that of *K. kingae*?

## 5. Regulation of *rtxA* Gene Expression by Phase Variation

It has long been known that bacteria can undergo phase variation through high-frequency reversible ON–OFF switching of gene expression that generates phenotypically divergent bacterial populations [105,106,107]. Phase variation arises through chromosome replication errors that alter the number of short tandem repeats and introduce frame shifts into open reading frames or affect gene promoter functions [106]. This facilitates evasion of host immunity and bacterial adaptation to diverse host environments [106]. Indeed, many bacterial pathogens use phase variation to control the production of flagella, fimbriae, pili, and other highly immunogenic virulence factors [108,109].

Over time, researchers discovered phase-variable methyltransferases associated with the type III restriction-modification (R-M) system that protects bacteria from foreign DNA [110,111]. The type III R-M system consists of methyltransferases that modify bacterial DNA and restriction endonucleases that cleave foreign DNA [112]. Some bacterial pathogens contain the type III R-M system methyltransferase genes with varying numbers of DNA repeats, which can be present in the ON and OFF forms, and the genes encoding restriction endonucleases that are silenced [106]. Experiments with a *Haemophilus influenza* knockout of the methyltransferase (*mod*) gene revealed the up- or downregulation of 16 genes that could be divided into 2 categories [106]. The first group consisted of genes encoding transport proteins and the second comprised genes for the heat shock proteins such as HtpG, DnaK, and GroEL involved in response to stressful environmental cues. When the *mod* gene was in the ON (in frame) form, proteosynthesis of transport proteins increased; the opposite was true for the OFF form. Thus, it was suggested that bacteria can use phase-variable methyltransferases to change their phenotype between two independent environments within the host [106]. The demonstration that phase variation of a single gene causes a change in the expression of an entire group of independent genes through epigenetic regulation in bacteria coined the term ‘phasevarion’ for a phase-variable regulon [106,112]. Later, epigenetic regulation by the phasevarion has also been demonstrated in other bacteria such as *N. meningitidis*, *N. gonorrhoeae* [113], *Helicobacter pylori* [114,115], and, recently, also in *K. kingae* [116].

The *mod* genes can be present in different alleles that encode different methyltransferases that can methylate diverse DNA sequences. Therefore, individual bacterial strains may regulate different sets of genes, which can lead to a largely heterogeneous population within a single bacterial species [112]. According to current knowledge, *K. kingae* has two active *mod* alleles, *modK1* and *modK2*, whose phase-varying activities are regulated by the actual number of 5′-AGCC-3′ repeats [116]. When the number of repeats is 13, the *modK* allele is ON (in frame for expression), and when it is 12, the *modK* allele is OFF (out of frame). The *groEL* and *dnaK* genes, which encode heat shock proteins, and the *rtxA* gene are controlled by the *modK1* allele. Switching to *modK1* ON phase yields an increased production of RtxA, and the attenuation of the production of GroEL and DnaK and the opposite occurs in the *modK1* OFF phase (Table 2) [116].

Considering that these results are consistent with operation of the phasevarion of *H. influenza*, it was hypothesized that *K. kingae* uses epigenetic regulation dependent on phase variation of the *modK1* gene to adapt to different environments within the host [116]. Indeed, the comparison of survival of *K. kingae* strains in *modK1* ON and *modK1* OFF phases at 46 °C revealed that the strain in the *modK1* OFF phase, having the heat shock protein expression upregulated, resisted the elevated temperature better [116]. Moreover, 21% of the surviving strains in the *modK1* ON phase switched the gene to the OFF form after 90 min of exposure to the elevated temperature. In addition, the ModK1 methyltransferase was observed to affect the proinflammatory response of human THP-1 macrophages. Exposure to the *K. kingae* strain in *modK1* OFF phase yielded increased release of interleukin-1β (IL-1β), IL-8, and tumor necrosis factor (TNF) by macrophages (Table 2) [116]. Since heat shock proteins can activate the immune system and the production of cytokines themselves [117], the increased expression of cytokines in this case could be due to the increased levels of DnaK and GroEL proteins [116].

## 6. General Structural Features of RtxA and Other RTX Toxins

Since only limited experimental data on the structure and function of RtxA are available, results obtained with more thoroughly studied RTX toxins can improve our understanding of the role of RtxA in the pathogenesis of *K. kingae* infections. Therefore, below, we summarize the results accumulated over the past few decades for some of the best-studied RTX toxins and discuss them in relation to RtxA.

RtxA and other RTX toxins are relatively large bacterial molecules with molecular masses between ~100 and 200 kDa (RtxA, 105 kDa; HlyA, 110 kDa; CyaA, 177 kDa) and consist of single polypeptide chains lacking cysteine residues. These toxins differ from other pore-forming toxins (PFTs) by the presence of several characteristic structural and functional domains and segments (Figure 1) [29,30,118,119,120,121]. The N-terminal part of each RTX toxin molecule contains a hydrophobic pore-forming domain with several putative transmembrane α-helices that can insert into the host cell membranes and can form cation-selective membrane pores. The central portion of the molecule comprises two conserved lysine residues that are post-translationally modified by a co-expressed acyltransferase that converts each RTX protoxin into an active toxin. The C-terminal part of the molecule contains a typical RTX domain comprising between ~10 and 40 characteristic glycine- and aspartate-rich nonapeptide repeats. The binding of calcium ions to these repeats is critical for the proper folding of the RTX domain into the characteristic β-roll structure and folding of the entire RTX toxin into its cytotoxic form. The C-terminal end of the molecule contains an unprocessed secretion signal that is required for the export of the RTX toxin from the bacterial cytosol through the T1SS directly into the external milieu.

The only exception to the arrangement of typical RTX toxins is the CyaA toxin, in which an enzymatic adenylate cyclase (AC) domain is fused to the N-terminus of the RTX hemolysin via a specific linker (Figure 1) [122,123].

### 6.1. N-Terminal Part with Pore-Forming Domain

The immediate N-terminal protein segments of RTX toxins, consisting of putative amphipathic α-helices, share relatively little sequence identity [118,124,125,126]. The HlyA mutants with a deletion of residues 10–19 or even 9–37 in the N-terminus are hemolytic on erythrocytes and are able to form active pores in planar lipid membranes composed of asolectin [124]. The HlyAΔ9–37 mutant even showed a 2.5-fold higher lytic capacity on sheep erythrocytes than the intact toxin [127]. Therefore, it was hypothesized that the N-terminal segment of HlyA is not the key region for pore formation, but may act as its regulator and/or be involved in the interaction of the toxin with the cell membrane [125,127,128,129].

The hydrophobic pore-forming domain of RTX toxins is located immediately downstream of the N-terminal segment (Figure 1) and consists of several putative transmembrane α-helices. It inserts into the membrane of a target cell and forms an ion-permeable pore that allows bidirectional ion fluxes leading to the colloid-osmotic (oncotic) lysis of the cell [32,34,130].

To identify the regions involved in pore formation by HlyA, three hydrophobic segments were deleted between residues 238–259, 299–327, and 366–410 of the molecule [127]. The HlyA mutant with the deletion of the first segment was only slightly active on bovine erythrocytes and showed increased conductivity in planar lipid bilayers without forming defined pores. Deletion of the second or third segment completely abolished the pore-forming activity of the HlyA mutants, but their stability and secretion were not altered. Moreover, deletion of the single polar aspartate residue (D243) within the first hydrophobic segment of HlyA substantially reduced the ability of the HlyA variant to form membrane pores, whereas substitution of the D243 residue with a glutamine or asparagine residue had little effect on pore formation [124]. A later report showed that residues 177–238 of HlyA are also involved in the pore-forming activity of the toxin [131]. In addition, cysteine scanning mutagenesis of HlyA identified a putative α-helix between residues 272–298 that may line the membrane pore formed by the toxin [126]. Moreover, the double substitution G284P + I287P completely abolished the hemolytic activity of the HlyA mutant, whereas binding to erythrocytes was not affected, indicating the importance of the G284 and I287 residues for the pore-forming activity of HlyA [126]. All these data suggested that residues 177–410 of HlyA form the pore-forming domain and play a key role in membrane pore formation [124,127]. Based on some sequence homology between HlyA and RtxA (~44%), it can be assumed that RtxA segments homologous to the analyzed hydrophobic segments of the pore-forming domain of HlyA are involved in the formation of RtxA pores, but this needs to be confirmed experimentally.

The pore-forming domain of CyaA is located between residues ~500 and 700 and consists of 5 hydrophobic α-helical segments predicted by the algorithm of Eisenberg between residues 502–522, 529–549, 571–591, 607–627, and 678–698 **[132,133,134,135,136]**. The deletion of the residues 623–779 within the pore-forming domain substantially reduced the hemolytic (pore-forming) activity of the CyaA mutant (by 88%) and also abolished its ability to translocate the enzymatic AC domain across the cell membrane (cell-invasive AC activity) of erythrocytes [50]. Several other reports showed that the first four predicted transmembrane α-helices of CyaA are involved in both the pore-forming and cell-invasive AC activity of the toxin, while the fifth predicted α-helix was exclusively involved in the pore-forming activity of CyaA [134,135,136,137,138,139].

The first suggestion that the pore-forming activity of RTX toxins may lead to cation fluxes across the cell membrane was provided in 1983, when HlyA was found to promote the accumulation of calcium ions and rapid depletion of potassium ions in erythrocytes [31]. Several later reports confirmed that the pores formed by RTX toxins are cation-selective [34,35,36,135,140]. Interestingly, we demonstrated that charge reversal by the E509K and/or E516K substitutions strongly reduced the cation selectivity of membrane pores formed by CyaA, suggesting that the residues E509 and E516 are located within or close to the membrane pore [134]. Moreover, the negative charge of the residue E570 was also necessary for the cation selectivity of the CyaA pore, suggesting its role as an ion filter [135]. Replacing the potassium ion with a less mobile lithium ion in experiments with RtxA on a black lipid membrane (BLM) showed an effect on the conductivity of the pore, supporting the preference of the pore for different cations [36]. Remarkably, HlyA and LtxA were also shown to enable ATP release from human erythrocytes directly through the membrane pores formed by the toxins [141], whereas the CyaA pores appear to be too small for ATP leakage [56].

### 6.2. Acylated Segment and Its Posttranslational Modification

The acylated segment of RtxA is located between the pore-forming domain and the RTX domain of the molecule, similarly as the acylated segments of the other RTX toxins (Figure 1). It was shown that RtxA and the other RTX toxins are produced as inactive protoxins (proRTXA) that are post-translationally acylated by co-expressed toxin-activating acyltransferases (RTXC) [37,142,143,144,145].

As first shown for HlyA, the proHlyA protoxin is acylated by its cognate acyltransferase HlyC, which uses acyl carrier protein (ACP) as an acyl chain donor [142,143,146]. The HlyC-modified HlyA toxin bears amide-linked acyl chains at the ε-amino groups of two conserved internal lysine residues, K564 and K690 [47]. In uropathogenic isolates of *E. coli*, the K564 and K690 residues of HlyA were mostly acylated by myristoyl chains (C14:0; ~68%), and the remaining acyl chains were identified as the very rare odd-carbon pentadecanoyl and heptadecanoyl chains (C15:0; ~26% and C17:0; ~6%, respectively) [147]. Later, we showed that recombinant HlyA co-expressed with HlyC in the *E. coli* strain BL21 was predominantly acylated by the C14:0 and hydroxymyristoyl (C14:0-OH) chains at the K564 (~84%) and K690 (~93%) residues, and only partially acylated by other acyl chains, such as lauroyl (C12:0), hydroxylauroyl (C12:0-OH), palmitoyl (C16:0), and palmitoleyl (C16:1), but not by the C15:0 and C17:0 chains [148]. It suggested that the uropathogenic and BL21 strains of *E. coli* likely differ in the composition of the acyl-ACP pool [148].

In contrast to HlyA, CyaA was initially found to be modified by the acyltransferase CyaC at the K983 residue by the C16:0 chain [52]. Later, when CyaA was overproduced in *B. pertussis* 18323, the C16:0 chain was also found attached at the K860 residue [54]. When CyaA was co-expressed with CyaC in *E. coli*, the recombinant CyaA toxin was mostly modified by the C16:0 (K860, ~46% and K983, ~22%) and C16:1 (K860, ~44% and K983, ~56%) chains, while only a very low proportion of the C14:0 chain was observed [139,149,150,151]. On the other hand, we found that the C14:0 chain is the major modification of the K558 (~18%) and K689 (~71%) residues of the recombinant RtxA cytotoxin co-expressed with RtxC in *E. coli* [37]. The remaining RtxA molecules were modified by the C14:0-OH (K558, ~5% and K689, ~18%), C12:0 (K689, ~2%), and C16:1 (K689, ~8%) chains [37].

RTXC acyltransferases are highly conserved among different bacterial genera, and some of them can activate heterologous RTXA protoxins [152,153,154,155]. For example, *A. pleuropneumoniae* hemolysin ApxIA heterologously acylated by HlyC exhibited hemolytic activity on erythrocytes, similarly as HlyA acylated by ApxC [152,155]. Analogously, *M. haemolytica* leukotoxin LktA heterologously activated by CyaC or HlyC exhibited the same activity and host cell specificity as LktC-acylated LktA [153,154]. However, the activation was not reciprocal because CyaA or HlyA co-expressed with LktC in *E. coli* were neither cytotoxic nor hemolytic [153,154]. To investigate why some RTXA protoxins are efficiently cross-activated by heterologous RTXC acyltransferases and others are not, we recently analyzed the acylation of CyaA, HlyA, and RtxA, each activated by one of the three acyltransferases CyaC, HlyC, or RtxC, respectively [148]. To exclude a possible influence of differences in the composition of the acyl-ACP pools of the original producer bacteria, the RTXA protoxins were co-expressed with the RTXC acyltransferases in the *E. coli* B strain BL21. Intriguingly, we found that each of the three acyltransferases specifically selected from the *E. coli* pool of acyl-ACPs the acyl chains of a specific length (C14 versus C16) for covalent attachment to the proRTXA protoxins. Moreover, the acyltransferases also determined whether only one or both of the two conserved internal lysine residues of the protoxins will be recognized and acylated. The CyaC acyltransferase preferentially used the C16 (C16:0 and C16:1) chains and functional assays showed that CyaA has to be acylated by these C16 chains to be active on target cells. In contrast, the HlyC and RtxC acyltransferases selected from the same acyl-ACP pool of *E. coli* preferentially the C14 (C14:0 and C14:0-OH) chains. Interestingly, HlyA exhibited biological activity when it was acylated both by the C14 chains by the action of HlyC and RtxC, as well as by the C16 chains attached by CyaC. However, RtxA was activated exclusively by the C14 chains [148]. RtxA acylated with the C16 chains by CyaC was impaired in the lysis of erythrocytes and showed very low overall membrane activity on planar lipid membranes [148]. It suggested that C16-acylated RtxA was impaired in binding and/or insertion into the membrane and/or in the ability to form membrane pores. However, the residual pores formed by C16-acylated RtxA exhibited similar properties to the membrane pores formed by C14-modified RtxA [148]. In this respect, C16-acylated RtxA resembles unacylated proRTXA protoxins, which exhibit low overall membrane activity, but when inserted into the membrane, they form pores with similar characteristics as acylated RTX toxins [37,133,156,157]. In summary, our results revealed for the first time that the RTXC acyltransferases select the adapted fatty acyl chains of specific lengths for activation of the RTX protoxins and that a structural and functional adaptation to the appropriate length of the attached acyl chains linked to the conserved lysine residues occurred in the RTXA toxins [148].

Although the activation by posttranslational acylation of RTX toxins has been shown to be essential for their cytotoxic activities [37,47,62,144,145,157], the precise molecular mechanisms by which the acyl chains confer activity to RTX toxins are still poorly understood. It has been shown that the acyl chains covalently linked to CyaA play an important structural role in folding of the toxin molecule into a biologically active conformation [158,159]. Moreover, the acyl chains were important for irreversible and productive interaction of CyaA with cells expressing its receptor, the integrin CD11b/CD18 [157,160]. The acyl chains linked to HlyA have further been shown to be required for irreversible insertion of the toxin into the target membrane [161] and for oligomerization of the toxin in membrane microdomains [162].

### 6.3. Calcium-Binding Repeat Domain

**Figure 5:**
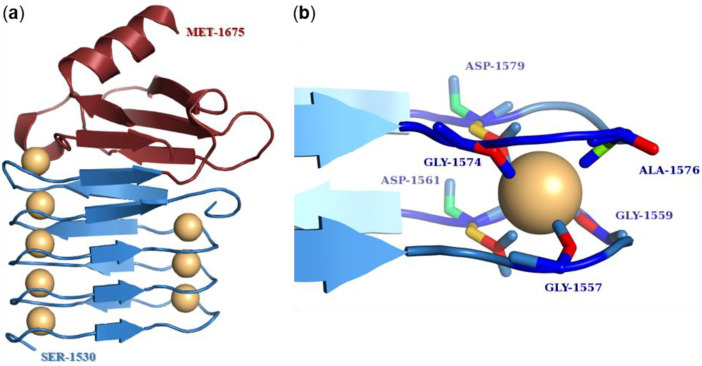
The crystal structure of residues 1530–1675 of CyaA. (**a**) The parallel β-roll structure of block V of the RTX domain and the capping structure of CyaA (PDB ID 6SUS). The blue color represents the nonapeptide repeats of block V, the red color represents the capping structure, and the calcium ions are shown in light orange. (**b**) A detailed view of the calcium-binding site within block V of the RTX repeats. Each nonapeptide motif forms two half-sites for calcium binding, with each calcium ion bound at a hexa-coordinated site between two consecutive turns. The residues whose side chains directly coordinate the calcium ion are highlighted.

The calcium-binding repeat (RTX) domain is located in the C-terminal part of the toxin molecule (Figure 1) and consists of characteristic glycine- and aspartate-rich nonapeptide repeats. These were at the origin of the historical name of the entire RTX protein family, where RTX stands for Repeats in ToXin [118]. The consensus motif of the repeat sequence is G-G-X-G-X-D-X-U-X, where X represents any amino acid residue and U represents the hydrophobic residue isoleucine, leucine, or valine [163,164]. The number of tandemly arranged nonapeptide repeats in the molecule varies from 10 to more than 40 in the different RTX toxins [29]. However, the repeat sequences of many RTX toxins only partially match the consensus sequence, so the exact number of repeat sequences in RTX toxins depends on how strictly the consensus motif is followed [164,165]. In 1988, Ludwig et al. hypothesized that the predominance of glycine- and aspartate-rich repeats might lead to a secondary structure characterized by β-turns [124]. These turns would allow negatively charged aspartate residues to create calcium-binding sites [124,166]. This conjecture was not far from the truth, as first shown by the crystal structure of the RTX domain of the alkaline protease of *Pseudomonas aeruginosa* and later confirmed by the X-ray structure comprising the last RTX repeat block of CyaA [163,167,168]. These crystal structures showed that the first six residues of the repeat sequence (G-G-X-G-X-D) form a β-turn involved in calcium binding, while the remaining three residues of the repeat (X-U-X) form a short β-strand (Figure 5a) [163,167,168]. Two consecutive repeats then form a complete repeat of a β-roll structure, and the hydrophobic U residues form a hydrophobic core [163,169]. The calcium ion binds between two adjacent β-turns through the negatively charged aspartate residues and carbonyl backbone groups of the residues forming a hexa-coordinated binding site (Figure 5b) [163,167,168,170,171,172]. Many reports have shown that the binding of calcium ions to the repeats triggers large conformational changes in the RTX toxin molecule that both expose specific peptide surfaces and convert an inactive form of the toxin into an active parallel β-roll structure [129,167,171,173,174,175]. Indeed, it is well-known that RTX toxins require calcium ions for their biological activity [37,173,176,177] and that calcium ions cannot be replaced by most divalent ions without reducing toxin activity [178,179].

In the absence of calcium ions, the RTX domain is inherently disordered, unstable, and highly hydrated. In fact, each RTX toxin molecule in the bacterial cytoplasm adopts a disordered structure called the premolten globule, with a high proportion of random turns and a low proportion of β-structures [172]. This structure is achieved by electrostatic repulsion of negatively charged aspartate residues and a low concentration of calcium ions and is preferred for protein secretion. The binding of calcium ions to the RTX domain upon secretion triggers the conversion of a disordered protein into a folded RTX toxin [171,172]. The folding of RTX toxins occurs simultaneously with protein secretion via the T1SS in a highly cooperative and vectorial manner from the C-terminus towards the N-terminus and is accompanied by dehydration of the RTX molecule [167,171,172,180].

The RTX domain of HlyA consists of 12–13 tandem nonapeptide repeats to which the binding of calcium ions is required for proper folding and activity of the toxin [124,166,180]. Indeed, an HlyA mutant lacking 11 of these repeats failed to bind calcium ions and was impaired in binding to erythrocytes [166,181]. Interestingly, HlyA folded in the presence of strontium ions, which have a similar ionic radius to calcium ions, exhibited complete hemolytic activity on erythrocytes, whereas the toxin activated with barium ions exhibited partial hemolytic activity [178]. Later experiments with proHlyA showed that the native conformations of the protoxin refolded in the presence of strontium and barium ions were virtually identical to the conformation of proHlyA folded in the presence of calcium ions [182]. On the other hand, magnesium ions were unable to drive the folding of proHlyA and activate the hemolysis of HlyA on erythrocytes [178,182]. This suggested that the nonapeptide repeats of HlyA can only bind cations with a specific geometry and size [182]. HlyA variants with deletions of different single nonapeptide repeats were still hemolytic on erythrocytes but required a higher concentration of calcium ions for hemolysis than intact HlyA [124]. However, the deletion of three or more repeats resulted in a complete loss of hemolytic activity of the HlyA mutants, even in the presence of high concentrations of calcium ions. Furthermore, the HlyA mutants were unable to compete with intact HlyA for binding to erythrocytes at low concentrations of calcium ions, but were able to form ion-permeable pores in planar lipid bilayers, even in the absence of calcium ions. These results suggested that the RTX domain of HlyA is responsible for the calcium-dependent binding of the toxin to the membrane of erythrocytes [124].

The RTX domain of CyaA is located between residues 1007 and 1613 and comprises 40–45 nonapeptide repeats, the exact number depends on the consensus criteria [49,165,180]. The repeats are arranged in 5 blocks consisting of 8–10 nonapeptides, and each block is flanked by linkers that are 23–49 residues long [170,180,183]. This organization of the RTX domain is unique to CyaA [180]. The C-terminal linker of each block of repeats is essential for calcium responsiveness and proper folding [167,170,184]. The amino acid segment 1166–1281 comprising the linker between the second and the third block of the RTX domain is critical for the binding of CyaA to its specific receptor on the surface of eukaryotic cells, the integrin CD11b/CD18 [57,160,185]. However, a CyaA mutant lacking residues 1245–1273 within this segment still exhibited at least partial invasive AC and hemolytic activities (~25%) in CD11b/CD18-negative erythrocytes [165]. The NMR structure of repeat block V, including residues 1530–1630, was recently solved and showed that it adopts a ‘hatchet head’-like structure with an N-terminal ‘blade’-like β-roll [167]. In addition, the C-terminal flanking region of block V located between residues 1631–1680 is absolutely required for calcium-induced folding of the molecule, as the CyaA mutant lacking this segment cannot bind calcium ions [184]. The nonapeptide repeats of CyaA are highly selective for calcium ions, similarly as the repeats of other RTX toxins [179,182]. When the Ca^2+^ ions were replaced by La^3+^, Ni^2+^, Cd^2+^, Mn^2+^, Ba^2+^, Zn^2+^, and Co^2+^, the activity of CyaA was significantly decreased. Of these ions, the toxin showed the greatest activity when manganese ions were used, but even that was only 11% activity compared to calcium ions [179,184].

No work has yet been published describing the RTX domain of RtxA, but it is very likely that its structure is highly similar to that of HlyA and other RTX toxins in that it forms a calcium-loaded β-roll structure.

### 6.4. C-Terminal Secretion Signal

The C-terminal end of each RTX toxin harbors the secretion signal (Figure 1) required for the initial recognition of the T1SS [29,186,187,188]. The presence of a topogenic sequence at the C-terminal end of the molecule is quite exclusive and one of the characteristic structural features of all members of the RTX protein family [29].

It was shown that the secretion of the 23 kDa C-terminal segment of HlyA (~210 residues) was as efficient as the secretion of intact HlyA, providing direct evidence for the C-terminal localization of the secretion signal [186]. In later studies, the localization of the secretion signal was narrowed down to the last 113 and later 53 residues of HlyA [189,190]. Interestingly, the fusion of the last 27 residues of HlyA with the *E. coli* membrane protein OmpF, which lacks its own N-terminal signal sequence, still resulted in detectable secretion of the chimeric construct into the medium [189].

Using several truncated variants of CyaA, it was shown that the C-terminal secretion signal of the toxin is located in the last 75 residues of the molecule [187,188,191]. Interestingly, the partial secretion of CyaA variants with deletion of the last 75 or 217 residues was observed, suggesting that the toxin contains at least 2 additional secretion signals that can be recognized by the T1SS [188]. One of these was located in the RTX domain between residues 1587 and 1631, suggesting that nonapeptide repeats of CyaA may be recognized as alternative C-proximal secretion signals by the T1SS [188]. A later study showed that a short segment between residues 1631 and 1647 contributed substantially to the secretion of CyaA, whereas the last 24 residues of the molecule were not important for toxin transport [191].

In 1985, it was shown that HlyA can be excreted from *E. coli* without proteolytic cleavage and cell lysis, and the same was later observed for CyaA, suggesting that the C-terminal secretion signal is not processed when the RTX toxins are exported to the extracellular environment [41,188]. Surprisingly, the primary sequences of the C-terminal secretion signals are the least conserved regions of RTX toxins, making it rather difficult to define a consensus sequence necessary for recognition by the secretion apparatus [83,118,188,192,193]. Nevertheless, it was shown that the HlyBD/TolC proteins can heterologously secrete the CyaA toxin, suggesting that the RTX transport proteins and the RTX toxins may be functionally interchangeable and that the different secretion signals of the RTX toxins must be somehow structurally conserved [118,187,188]. Indeed, the C-terminal secretion signals appear to be determined by conserved elements of secondary and perhaps even tertiary structural features [119,192,194]. Mutational analysis of the C-terminal region of HlyA revealed that the secretion signal is located in the last 48 residues and comprises 3 functional segments: an amphipathic and charged α-helix followed by a 13 residue-long uncharged region and an 8 residue-long hydroxylated tail in the outermost part of the C-terminus of the HlyA molecule [192]. Analogous segments were found in the C-terminal sequences of other RTX toxins secreted by the T1SS [192]. A later prediction of the C-terminal secretion signal of HlyA proposed that it consists of two amphipathic helices linked with a sequence of 8–10 residues. While the first amphipathic helix and the linker segment were important for efficient transport of the toxin, the second helix was not essential for transport [194].

In 2016, the NMR structure of the C-terminal assembly of CyaA and its role in protein folding was revealed [167]. Immediately after translocation through the T1SS, the C-terminus of CyaA forms a capping structure that is essential for the highly cooperative binding of calcium ions and stacking of the last RTX block β-roll structure, thus driving the C-to-N-vectorial folding of the entire toxin molecule [167,184]. The C-terminal capping structure and the subsequently formed β-roll also form a Brownian ratchet that prevents the backsliding of the translocated toxin in the channel-tunnel assembly of the T1SS conduit that spans the bacterial cell envelope [167,171]. A similar C-terminal capping structure is required for the calcium-dependent folding and biological activity of HlyA, LtxA, and ApxIA, suggesting that the capping structure is a common structural and functional feature of all RTX toxins [167]. Moreover, mutations within the last six residues of HlyA did not affect the secretion of the toxin, but its hemolytic activity was significantly reduced due to an altered overall folding of the molecule [195].

To date, no work of this type has been published for RtxA, but it can be assumed that the C-terminal secretion signal of RtxA has similar structural features as the secretion signals of other RTX toxins.

### 6.5. Adenylate Cyclase Domain and Linker Segment of CyaA

Unlike other RTX toxins, CyaA contains an N-terminal adenylate cyclase (AC) enzyme domain (~400 residues) fused to the C-terminal RTX hemolysin moiety by a so-called ‘AC-to-Hly’ linking segment (residues ~400–500; Figure 1) [83,196]. The AC domain is delivered to the cytosol of a target cell by the RTX hemolysin moiety, which enables the binding of CyaA to the cell surface [50,122,160,197,198]. Inside cell cytosol, the AC enzyme is activated by binding of calmodulin and catalyzes unregulated conversion of ATP to supraphysiological concentrations of cAMP, thereby subverting cellular signaling [122,123,199]. In parallel, the RTX hemolysin moiety forms small cation-selective membrane pores that cause colloid-osmotic cell lysis and contribute to the cytotoxicity of CyaA in vitro [122,133]. The increase in intracellular cAMP levels due to the catalytic activity of the AC domain of CyaA begins almost instantaneously after the toxin is added to sheep or human erythrocytes [51,200]. The cAMP concentration doubles within 1 min and reaches its maximum within 20–40 min [51,200]. However, the hemolytic activity of CyaA has a lag phase of 40–80 min and reaches its maximum after 4 h of incubation [51]. These two distinct activities of CyaA can be separated, and the balance between them can be shifted by changes in temperature, free calcium ion concentration, acylation status of the toxin, or specific substitutions in the pore-forming domain [35,134,135,149,201,202]. In addition, a CyaA mutant lacking the AC domain exhibited the same hemolytic activity on erythrocytes as the intact toxin [197]. All these findings suggested that the AC domain and the RTX hemolysin moiety of CyaA are functionally independent and that two distinct conformers of the toxin exist. One would be involved in AC enzyme translocation into cells and the other would account for assembly of the oligomeric pore accounting for the pore-forming (hemolytic) activity of CyaA [122,123,134,203].

Limited proteolysis with trypsin revealed that the calmodulin-bound AC domain consists of two trypsin-resistant subdomains, one of 25 kDa (T25; residues 1–224) and the second of 18 kDa (T18; residues 225–399), which remained associated with calmodulin in a catalytically active ternary complex [204]. In the absence of calmodulin, the AC domain was completely inactivated with trypsin in <3 min [204]. Later, a crystal structure of the AC domain with the C-terminal domain of calcium-loaded calmodulin was published, showing four discrete regions of the AC domain interacting with the calmodulin molecule, as well as the catalytic site located between the T25 and T18 subdomains [205].

The membrane-interacting AC-to-Hly linking segment of CyaA is essential for translocation of the AC domain across the cell membrane and regulates the pore-forming (hemolytic) activity of the toxin [196,206]. The recent NMR structure of the AC-to-Hly linking segment in dodecylphosphocholine micelles showed that it consists of two alpha helices, one of which is hydrophilic and one hydrophobic [207]. Site directed mutagenesis revealed that two clusters of negatively charged residues (E419 to E432 and D445 to E448) within the AC-to-Hly linking segment regulate the equilibrium between the AC domain translocating and pore-forming activities of the toxin [207]. Moreover, it was recently proposed that the segment comprising residues 454–484 of CyaA penetrates the cell membrane and binds cytosolic calmodulin, thereby triggering translocation of the N-terminal AC domain across the membrane [208].

## 7. Secretion of RtxA and Other RTX Toxins

**Figure 6:**
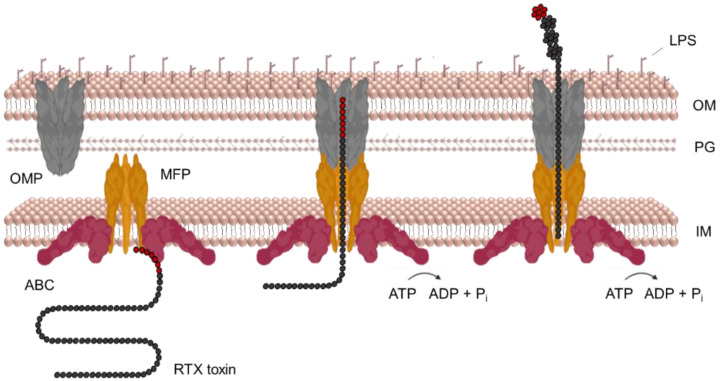
Schematic representation of the T1SS assembly process. Recognition of a C-terminal secretion signal of RTX toxin by the proteins ABC and MFP is followed by recruitment of the trimeric TolC complex and formation of the transport channel. The secretion channel spans both membranes of the Gram-negative bacterium and secretes the toxin from the cytosol into the extracellular environment in a one-step mechanism. The energy for secretion is provided by ATP hydrolysis. ABC, ATP-binding cassette transporter; MFP, membrane fusion protein; OMP, outer membrane protein; LPS, lipopolysaccharide; OM, outer membrane; IM, inner membrane; PG, peptidoglycan. This image was created using BioRender.com (accessed date: 22 February 2022).

The T1SS apparatus of Gram-negative bacteria transports various proteins in size and function, including RTX toxins [209]. The T1SS consists of three proteins: (i) an ATP-binding cassette (ABC) transporter; (ii) a membrane fusion protein (MFP); and (iii) an outer membrane protein (OMP) of the TolC protein family. The ABC transporter is anchored in the cytoplasmic membrane and binds to the MFP component, which has a large periplasmic domain in addition to the transmembrane segment. The OMP component interacts with the outer membrane of bacteria and spans across a large portion of the periplasmic space. The initial recognition of an uncleaved C-terminal secretory signal of RTX toxin by the ABC transporter and MFP triggers the assembly of a functional trans-envelope complex through further specific interactions of MFP and OMP in the periplasm. This transport complex bypasses the periplasm and releases the toxin directly into the extracellular environment in a single step mechanism (Figure 6) [190,209,210,211,212,213,214]. Interestingly, the function of the ABC and MFP proteins is unique to the T1SS, whereas TolC is pleiotropic and has a variety of other functions, such as efflux of toxic molecules [215,216].

The secretion of HlyA by *E. coli* is the best studied among RTX toxins, and for this reason, the paradigm of the T1SS mechanism is mainly based on its analysis. No work has yet been published describing the secretion of RtxA via the T1SS, but we assume that it may be very similar to that of HlyA.

The three genes *hlyB*, *hlyD*, and *tolC* express the transporter proteins required for secretion of the HlyA toxin [82,143,217,218]. The *hlyB* and *hlyD* genes are located at the same locus as the *hlyA* and *hlyC* genes, but the tolC gene is located elsewhere in the genome (Figure 2). The *hlyBD*/*tolC* genes are transcribed constitutively so that the bacteria always have the proteins to assemble the secretory apparatus. However, the *hlyA* and *hlyC* genes are inducible and their transcription occurs only in the mid to late exponential phase of growth of uropathogenic *E. coli* strains [82,143,217,218,219].

The ABC transporter of uropathogenic *E. coli* called HlyB is a 77 kDa protein located in the inner bacterial membrane [212,220] and is thought to be active as a homodimer [209,212,217,218,220]. HlyB consists of an N-terminal cytoplasmic domain, a transmembrane domain (TMD) that anchors the protein in the inner membrane, and a C-terminal nucleotide binding domain (NBD) in the cytoplasm [209,221,222]. While the TMD and NBD are typical of ABC transporters [209,223], the N-terminal end of HlyB contains an additional 123 residue-long extension located in the cytoplasm. Because of its 42% homology to the C39 peptidase, this segment has been termed the C39-like domain (CLD). Interestingly, the CLD has no catalytic activity but is essential for toxin secretion as it binds the unfolded RTX domain of HlyA [216,222].

A topological model of HlyB predicted that the TMD contains six to eight transmembrane segments spanning the inner membrane, most likely α-helices. It is believed that the function of the TMD is to bind HlyA and facilitate its transport across the inner bacterial membrane [224,225]. The function of the NBD is to provide energy for the secretion process by binding and hydrolyzing ATP molecules [210,226,227,228]. ATP hydrolysis is the crucial energy source for the transport of HlyA [213,229,230,231]. When an inhibitor of ATP synthase (2,4-dinitrophenol) was used, the secretion of HlyA was completely stopped [229]. Furthermore, mutations in the NBD that altered ATP hydrolysis did not prevent the assembly of the secretory complex but inhibited the secretion of HlyA [213]. However, the use of proton motive force (PMF) for T1SS transport was also investigated [232]. The inhibition of PMF by the ionophore carbonyl cyanide m-chlorophenylhydrazone (CCCP) showed a direct effect on the onset of transport of the C-terminal HlyA polypeptide (22.4 kDa). In contrast, the inhibition of PMF a few minutes after the translation of this C-terminal polypeptide had no effect on its secretion [232]. Thus, PMF is probably used early in the secretory process, perhaps in the assembly of the secretory apparatus, but the main energy source for secretion of HlyA is ATP hydrolysis [229,230,231,232].

The X-ray structure of the NBD of HlyB (residues 467–707) was solved in 2003 [221]. It contains two domains, the catalytic domain arm I, where ATP hydrolysis occurs, and the signaling domain arm II, which is thought to be involved in cross-signaling between the NMD and TMD of HlyB [221]. Interestingly, the NBD of HlyB was also found to specifically recognize the C-terminal sequence (57 residues) of HlyA [228]. Moreover, the binding of ATP to the NBD facilitated dissociation of the NBD-HlyA complex and presumably initiated secretion by displacement of HlyA [228].

In general, ABC transporters contain Walker A motifs responsible for binding and subsequent ATP hydrolysis [223,233]. However, the NBD of HlyB also contains the Walker B motif in addition to the Walker A [221]. When these motifs interact, the ATP molecule cannot bind to the NBD, and this was thought to be the phase without HlyA. The binding of the C-terminal sequence of HlyA to the NBD would lead to conformational changes in the Walker motifs that allow ATP binding. This could possibly be the initiation mechanism of the secretion process by the T1SS [221,223].

The MFP of uropathogenic *E. coli* called HlyD (53 kDa) consists of three domains. A short N-terminal cytoplasmic domain (1–58), followed by a single transmembrane domain (59–80) and a large C-terminal periplasmic domain (81–478) [214,219,224,234]. Originally, HlyD was thought to be active as a trimer [213]. However, based on the recently obtained crystal structure of the periplasmic domain of HlyD, it was suggested that the protein is active as a hexamer [235,236].

The cytosolic N-terminal domain of HlyD contains a 25 residue-long amphipathic α-helix with a downstream segment of charged residues. This domain is not necessary for the assembly of the T1SS apparatus but is absolutely critical for the secretion of HlyA [214,219]. After binding to HlyB, HlyA also binds to HlyD. This binding leads to the recruitment of TolC and the formation of the transport channel through which the toxin is subsequently secreted. The recruitment of TolC appears to be controlled by the charged segment mentioned above. Therefore, the binding of HlyA to HlyB is not sufficient to trigger secretion, but must be accompanied by the binding of the C-terminal part of HlyA to HlyD [214].

The single transmembrane segment of HlyD anchors the protein in the inner bacterial membrane [219,237,238].

The periplasmic C-terminus of HlyD specifically interacts with the periplasmic domain of TolC and together they form a compact transport channel [213,236]. In 2016, the crystal structure of a large part of the C-terminal periplasmic domain (residues 96–372) was obtained. It consists of two parts, an α-helical domain and a lipoyl domain. The α-helical domain consists of three elongated α-helices with an α-helical tip region that has been shown by mutagenesis and cross-linking experiments to be required for specific interaction with TolC [236].

The HlyD protein is very stable in the T1SS apparatus. However, in the absence of TolC, the HlyB–HlyD complex becomes unstable and likely dissociates [219]. This suggests that the formation and dissociation of the T1SS complex is a dynamic process.

In 2000, the X-ray structure of TolC (OMP) from *E. coli* was solved, which contributed significantly to the current knowledge of the mechanism of T1SS-mediated secretion [239]. TolC (55 kDa) consists of two domains, a β-strand domain and an α-helical domain, and is active as a trimer. In the trimer complex, the four β-strands of each monomer associate to form a 12-stranded β-barrel channel anchored in the outer membrane. The β-barrel channel is wide open to the extracellular environment and fully accessible to the solvent [82,209,239,240]. Similarly, the four α-helices of each monomer associate to form a main body of the TolC structure, a channel in the periplasm composed of twelve α-helices [239]. The bottom of the α-tunnel is tightly closed with groups of coiled helices, preventing extracellular ions or molecules from entering the periplasm [239,241]. The entire channel conduit is 14 nm-long (β-barrel 4 nm, α-tunnel 10 nm) and has a diameter of 3.5 nm, which decreases towards the bottom [239].

The closed periplasmic end of TolC is extremely stable and opens only when the secretory apparatus is assembled by a specific interaction of TolC with the HlyB–HlyD complex [214,241]. The interaction between TolC and HlyD causes the coiled helices of TolC to untwist in an iris-like mechanism. This makes the interior of the α-domain of TolC available for the transport of HlyA [214,239,242,243].

The investigation of the electrophysiological properties of trimeric TolC in planar lipid bilayers revealed that it forms a stable cation-selective channel [241]. The cation selectivity is probably ensured by six conserved aspartate residues forming an aspartate ring at the entrance of the TolC channel [244]. The aspartate ring also affects the closure of the TolC entrance when the pH decreases and the carboxy groups of the aspartate residues become protonated. It prevents the repulsion of the residues and leads to the closure of the TolC entrance [241,244].

The C-terminal position of the secretion signal means that secretion can only occur once the translation of the protein has been completed [209]. The protein translation rate in exponentially growing *E. coli* cells would range from 12 to 17 residues per second, and the complete synthesis of the HlyA molecule would take between 60 and 80 s [245]. This is plenty of time for a partially translated protein to start folding. However, the RTX toxins are transported in an unfolded state [246]. This was demonstrated in experiments with a chimeric protein consisting of the signal-free maltose-binding protein (MalE) and the C-terminal segment of HlyA. The fusion protein was expressed in the cytosol of bacterial cells but was poorly secreted by the HlyBD/TolC T1SS because MalE was folding in the cytosol. However, when folding-disrupting point mutations were introduced, the secretion of the fusion protein was significantly increased [246]. This was confirmed by experiments involving a fusion of the enhanced green fluorescent protein (eGFP) to the N-terminus of the 218 C-terminal residues of HlyA [247]. The fusion protein could only enter the HlyBD/TolC T1SS but was unable to complete the secretion process due to the fast folding of eGFP and stalling inside the translocator [247]. Another evidence that HlyA is secreted in an unfolded state comes from the diameter of the TolC channel of only 3.5 nm, which would not allow passage of even a partially folded HlyA molecule [239,248].

The disordered state of the RTX toxin is controlled by the low concentration of calcium ions in bacterial cytoplasm [171,249]. As mentioned earlier, calcium ions are required for the folding of RTX toxins [171]. However, the intracellular calcium concentration in *E. coli* is about 170–300 nM, whereas a calcium concentration of about 1.5 mM (the physiological extracellular concentration) is required for the proper folding of HlyA [249,250]. Thus, the low concentration of intracellular calcium ions ensures that HlyA remains unfolded [171]. In the case of CyaA, it has also been suggested that electrostatic repulsion of the negatively charged aspartate residues plays a role in keeping the toxin in an intrinsically disordered state [172]. This raises the question of how the translated HlyA protein is protected from proteolytic degradation, which naturally occurs in the cytoplasm, in particular when the secretion process is thought to occur without the aid of a chaperone [182,223]. Nevertheless, this question has not yet been answered.

The secretion process of HlyA begins with the recognition of the C-terminal secretion signal by the NBD of HlyB. This results in conformational changes that allow ATP to bind to the NBD and initiate translocation of HlyA [228]. It is likely that HlyA next binds to the N-terminal cytoplasmic domain of HlyD through its C-terminal secretion signal [214]. It has also been shown that the RTX domain of HlyA binds to the CLD of HlyB. However, when this occurs in the process of secretion is unknown [222]. Binding of the C-terminal secretion signal of HlyA to HlyD triggers the recruitment of TolC, establishing the formation of a transient channel conduit from the cytosol to the extracellular environment [213,214]. The energy source for this step is probably PMF [232]. Thereafter, the secretion of HlyA through the translocon channel can begin. The exact mechanism of the translocation of HlyA through the T1SS remains unknown, but the energy is provided by ATP hydrolysis [210,226,227,228]. The secretion of HlyA is unidirectional, with the C-terminus first appearing outside the bacterial cell [247]. The rate of secretion of HlyA is approximately 16 residues per second, such that the entire toxin is secreted within approximately 70 s [251].

The folding of CyaA on the external bacterial surface is triggered by the binding of calcium ions. This process occurs simultaneously with the extrusion of the protein from the translocon upon exposure to extracellular calcium ions [167,171,172]. When the C-terminal portion of CyaA enters the extracellular environment, the first calcium ion is bound to CyaA via negatively charged aspartate residues and the carbonyl backbone [167,171,172]. This leads to the establishment of a π–π interaction between two aromatic residues (W1645 and Y1646 in CyaA) in the hydrophobic core of the resulting capping structure [167]. This was also observed for HlyA as the W914 residue was a key residue for subsequent folding of the RTX domain of HlyA [182]. The capping structure is subsequently folded and serves as a platform for the folding of RTX block V of CyaA [167]. Interestingly, the folding of the secreted C-terminal portion of CyaA is crucial for the entropic stabilization of the toxin [169]. In the subsequent vectorial calcium-driven folding of the RTX domain of CyaA, the C-terminal flanking segments of each repeat block play an important role [167,184]. The structure of the four linkers of the RTX blocks in the CyaA toxin appears to be highly similar [183]. In addition, each RTX block-linking fragment of CyaA contains a conserved aromatic residue. These aromatic residues are thought to be crucial for the proper folding of the adjacent block of RTX repeats [139,184,252]. In CyaA, the binding of extracellular calcium ions accelerates the secretion process by triggering the formation of intramolecular Brownian ratchets. This prevents the translocated CyaA toxin from backsliding in the translocation conduit, resulting in an increased rate of secretion [167]. However, this was not observed with HlyA toxin, and it remains possible that the Browning ratchet mechanism is exclusively related to the secretion of the substantially larger CyaA protein [251].

It has already been mentioned that proRTXA is activated by a posttranslational modification in the cytoplasm by the acyltransferases RTXC [37,142,143,144,145]. Interestingly, this activation is not necessary for the secretion of the RTX protein, since unacylated proHlyA was secreted as efficiently as acylated HlyA [253]. However, the post-translational modification of proRTXA is critical for folding of the RTX toxin outside of the bacterial cell [158,159]. It was shown that the acyl chains covalently bound to CyaA contribute to the folding of the toxin into a compact protein [159].

The export proteins HlyD and TolC may not only form the transport channel but also be involved in the final folding of HlyA. Mutational analysis of HlyD and TolC resulted in decreased hemolytic activity of secreted HlyA variants [238,254]. After the secretion of HlyA, TolC detaches from the HlyB–HlyD complex, which remains in the inner membrane and is ready for translocation of new substrates [213,214,255].

## 8. Interaction of RtxA and Other RTX Toxins with Target Cells

Based on cell type and species specificity, members of the RTX toxin family were originally divided into a group of hemolysins and a group of leukotoxins [29,30]. While the hemolysins exhibited toxicity on various cell types isolated from different mammalian species, the leukotoxins showed rather low cell type and species specificity. It was later found that the difference was due to the fact that hemolysins recognize cell surface structures that are widely expressed on virtually all mammalian cells (glycoproteins, glycolipids, cholesterol), whereas leukotoxins bind specifically to β_2_ integrins that are expressed exclusively on the cell surface of leukocytes [29]. However, some promiscuous RTX toxins originally classified as hemolysins (e.g., HlyA and CyaA) were later found to bind preferentially to β_2_ integrin-expressing leukocytes [48,53]. Similarly, some leukotoxins (e.g., LtxA and LktA) originally shown to bind to β_2_ integrins of leukocytes were found to exhibit detectable cytotoxic activity on β_2_ integrin-negative erythrocytes [61,69]. However, while RTX toxins bind to β_2_ integrin-expressing cells with high affinity and in a saturable manner, their binding to β_2_ integrin-negative cells is usually of low affinity and not saturable [53,57,256]. The following three subsections summarize in detail how RTX toxins interact with target cells via (i) specific β_2_ integrin receptors (Table 3), (ii) other cell surface structures (Table 3), and (iii) outer membrane vesicles.

### 8.1. Interaction with Specific β_2_ Integrin Receptors

**Figure 7:**
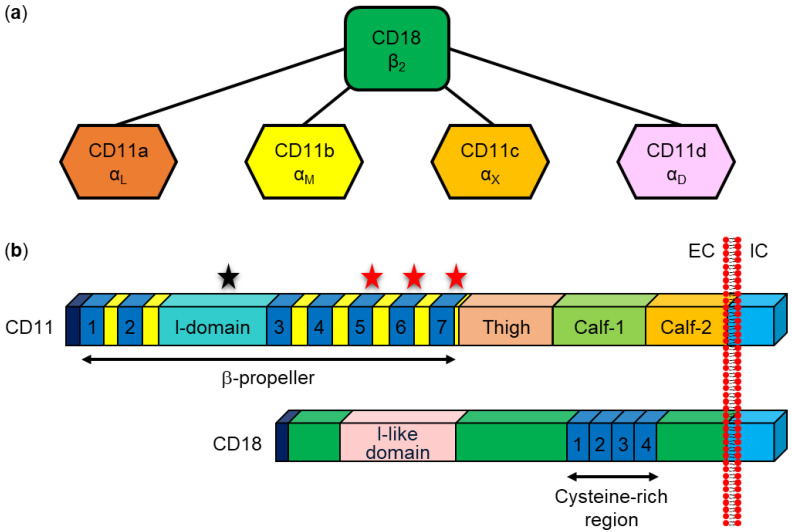
Schematic representation of the β_2_ integrins. (**a**) The common CD18 (β_2_) subunit (green) non-covalently associates with one of the four α-subunits, including CD11a (α_L_; orange), CD11b (α_M_; yellow), CD11c (α_X_; gold), and CD11d (α_D_; lavender). (**b**) The CD11 and CD18 subunits of β_2_ integrins consist of a long N-terminal extracellular domain, a single transmembrane α-helical segment, and a short C-terminal cytoplasmic segment. The extracellular domain of the CD11 subunits consists of an N-terminal secretion signal (navy blue), 7 β-sheet repeats (1 to 7; dark blue) that fold into a β-propeller domain, an I-domain (water blue) inserted between repeats 2 and 3 of the β-propeller domain, a thigh domain (sienna), and 2 calf domains (calf-1 in green and calf-2 in gold). The I-domain contains an Mg^2+^-binding site (black star) and repeats 5, 6, and 7 have Ca^2+^-binding EF-hand motifs (red stars). The extracellular domain of the CD18 subunit contains an N-terminal secretion signal (navy blue), an I-like domain (light pink), and a cysteine-rich region with four I-EGF-like domains (1 to 4; dark blue). Extracellular (EC) and intracellular (IC) compartments are separated by the cell membrane (red dots with short black lines).

Five RTX toxins, LtxA from *A. actinomycetemcomitans*, HlyA from *E. coli*, LktA from *M. haemolytica*, ApxIIIA from *A. pleuropneumoniae*, and CyaA from *B. pertussis*, have been shown to interact specifically with β_2_ integrins expressed exclusively on the surface of leukocytes (Table 3) [48,53,70,257,258]. The β_2_ integrins belong to an integrin superfamily consisting of 24 heterodimeric cell surface adhesion and signaling receptors that bind various soluble ligands, extracellular matrix ligands, and cell surface ligands [38,259,260]. The subclass of β_2_ integrins consists of four heterodimeric transmembrane glycoproteins with the same β_2_ subunit and four different α subunits (Figure 7): α_L_β_2_ (CD11a/CD18, or LFA-1), α_M_β_2_ (CD11b/CD18, complement receptor 3 (CR3), or Mac1), α_X_β_2_ (CD11c/CD18, p150/195, or CR4), and α_D_β_2_ (CD11d/CD18). The β_2_ integrins play an essential role in various leukocyte functions (trafficking, production of reactive oxygen species, phagocytosis, etc.) and deficiency in their expression or function results in a rare immunodeficiency syndrome known as leukocyte adhesion deficiency, characterized by increased susceptibility to the development of life-threatening bacterial and fungal infections [38,259,260,261].

**Table 3:** Interaction of RTX toxins with host cells via specific β_2_ integrin subunits and other cell surface structures.

RTX Toxin	β_2_ Integrin Subunit	Binding Site(s) on β_2_ Integrin Subunit	Ref.	Other Cell Surface Structures	Ref.
**RtxA**	None		[262]	– Cell surface oligosaccharides – Cholesterol	[37,262]
**HlyA**	CD18	NA ^1^	[48,263]	– Glycophorin – Cholesterol	[264,265,266]
**CyaA**	CD11b	– Residues 614–682 encompassing the C-terminal end of the β-propeller domain and the N-terminal part of the thigh domain	[53,57]	– Gangliosides – Cell surface oligosaccharides – Sphingomyelin – Cholesterol	[55,267,268,269,270,271,272]
**LtxA**	CD18	– I-EGF-like domains 2, 3, and 4 – Cytosolic domain	[48,64,263,273,274]	– Gangliosides – Sialic acid residues linked to glycosylated cell surface structures – Cholesterol	[63,65,275,276,277]
	CD11a	– β sheets 1 and 2 of the β-propeller – Cytosolic domain	[48,274,278,279]		
**LktA**	CD18	– Residues 5–17 in the signal peptide – Residues 1–291 – I-EGF-like domain 3	[70,280,281,282,283,284,285,286,287,288]	NA ^1^	
**ApxIIIA**	CD18	NA ^1^	[257]	NA ^1^	

^1^ NA: no data available.

The first RTX toxin observed to bind to β_2_ integrins was the LtxA leukotoxin from *A. actinomycetemcomitans* [48], which specifically kills human leukocytes and leukocytes from Old World primates [58,59,60]. In 1997, Lally et al. used a monoclonal antibody (mAb) that inhibited the cytolytic activity of LtxA on toxin-sensitive human leukemic HL-60 cells to immunopurify two polypeptide chains from detergent-solubilized cell membranes [48]. These polypeptides were identified by microsequencing as the alpha and beta subunits of the β_2_ integrin CD11a/CD18. To confirm the interaction between LtxA and CD11a/CD18, other anti-CD11a and anti-CD18 mAbs were used to demonstrate inhibition of LtxA-mediated cytotoxicity, with the highest inhibitory effect (~90%) observed with the anti-CD18 mAb KIM185. A direct binding experiment showed the ability of immobilized LtxA to bind the CD11a/CD18 integrin from cell lysates. Furthermore, LtxA-resistant human erythroleukemic K562 cells became sensitive to the toxin when transfected with the genes encoding human CD11a and CD18 [48]. Using K562 cells ectopically expressing human/bovine CD11a/CD18 heterodimers, it was later shown that the human CD18 subunit is the functional receptor for LtxA that confers species-specific sensitivity to the toxin [273]. Experiments with human/bovine CD18 chimeras then showed that residues 500–600, which contain the integrin epidermal growth factor (I-EGF)-like domains 2, 3, and 4 of human CD18 (Figure 7b), are essential for the sensitivity of the cells to the effects of LtxA [273]. It was later shown that K562 cells ectopically expressing the human β_2_ integrins CD11b/CD18 and CD11c/CD18 are similarly sensitive to purified LtxA as cells expressing human CD11a/CD18 [64]. However, in ligand blotting experiments, LtxA bound only to the CD18 subunit but not to the alpha subunits of the β_2_ integrins [64]. This confirmed previous work showing that the CD18 subunit harbors the major binding site for LtxA [64,273]. Experiments with knockout cells derived from the human monocytic cell line U937 that lacked individual subunits of the β_2_ integrins further confirmed that the CD18 subunit is necessary for the cytotoxic activity of LtxA, whereas all four alpha subunits were redundant for toxin activity [263]. Moreover, the genetic complementation of U937 cells lacking CD18 subunit expression with either intact CD18 or a CD18 variant lacking the cytoplasmic tail required for signaling restored LtxA sensitivity, suggesting that β_2_ integrin signaling was not required for LtxA cytotoxicity [263]. All these results demonstrated that the human CD18 subunit of β_2_ integrins is necessary for the binding and cytotoxic activity of LtxA [64,263,273].

Some reports suggest that the CD11a subunit may also be involved in the interaction with LtxA. Using human/murine CD11a chimeras, Kieba et al. showed that N-terminal β sheets 1 and 2 of the β-propeller domain of the human CD11a subunit (Figure 7b) are required for cell sensitivity to LtxA [278]. In addition, short peptides corresponding to the reported LtxA binding site in the β-propeller domain of CD11a blocked LtxA-mediated cytotoxicity in THP-1 cells by inhibiting the LtxA-CD11a/CD18 interaction [279]. In later work, labeled LtxA was used to show that the toxin enters cells in a CD11a/CD18-dependent manner and its translocated domain binds to and immobilizes the integrin via its cytosolic domains [274]. Fluorescence resonance energy transfer (FRET) microscopy using a cell line expressing fluorescently labeled cytosolic domains of CD11a/CD18 then showed that the internalized portion of LtxA acts on the integrin from the cytosolic side and brings the cytosolic domains of the CD11a and CD18 subunits closer together. Surface plasmon resonance measurements showed that LtxA has a strong affinity for the cytosolic domains of both the CD11a and CD18 subunits, whereas the affinity of the toxin for the cytoplasmic domains of the CD11b and CD11c subunits was significantly lower [274].

In the same publication, describing the interaction between CD11a/CD18 and LtxA, Lally et al. showed that CD11a/CD18 is also recognized by the HlyA toxin of *E. coli* [48]. The authors demonstrated that mAbs specifically recognizing the CD11a and CD18 subunits efficiently inhibit the cytolytic activity of HlyA in toxin-sensitive HL-60 cells and that K562 cells ectopically expressing CD11a/CD18 are more sensitive to HlyA than parental cells [48]. However, Valeva et al. later reported that HlyA binds nonspecifically to cells without requiring CD11a/CD18 or any other specific receptor [256]. The authors used radioactively labeled HlyA to show that the toxin binds to human granulocytes in a nonspecific and nonsaturable manner. Competition experiments in which radioactively labeled HlyA was incubated with granulocytes or erythrocyte ghosts in the presence of increasing amounts of unlabeled HlyA also failed to reveal the existence of any specific toxin receptors on cell membranes. Furthermore, the expression, blocking, or activation of the CD11a/CD18 integrin had no effect on the binding of HlyA to target cells [256]. This was later confirmed, when Munksgaard et al. demonstrated that K562 cells ectopically expressing CD11a/CD18 were similarly resistant to HlyA-induced lysis as parental K562 cells [65], which was in contrast to a similar experiment previously performed by Lally et al. [48]. To resolve these controversies, Ristow et al. performed an unbiased genome-wide positive selection in a mutant library of U-937 cells to identify host factors that contribute to HlyA cytotoxicity [263]. The selection results showed that the CD18 subunit was necessary and sufficient for the cytotoxic activity of HlyA, whereas all four alpha subunits were not required at all for the cytotoxic activity of the toxin. This was confirmed by a far-western blot, which showed that HlyA binds to the CD18 subunit but not to the alpha subunits of the β_2_ integrins. Furthermore, the genetic complementation of CD18-deficient cells with either intact CD18 or CD18 lacking the cytoplasmic tail restored cell sensitivity to HlyA, suggesting that β_2_ integrin signaling is not required for the cytolytic activity of HlyA [263]. These results suggest that the CD18 subunit of the β_2_ integrins serves as a receptor for HlyA.

The first report describing the interaction of LktA with the β_2_ integrins was published in 1998, when BL3 bovine lymphocyte cells were used to study the induction of apoptotic cell death by LktA [70]. The pretreatment of BL3 cells with anti-CD11a/CD18 or anti-CD18 mAb, but not anti-CD11b or anti-CD11c mAb, inhibited LktA-induced apoptosis. A pull-down assay with LktA-coated beads then indicated that the CD18 subunit of the β_2_ integrins is involved in binding of LktA to the surface of BL3 cells [70]. This observation was confirmed in later work showing that CD18 isolated from BL3 cell membranes binds LktA and that anti-CD11a/CD18 and anti-CD18 mAbs cause partial inhibition of LktA-induced cytolytic activity in BL3 cells [280]. Similarly, the direct binding of LktA to bovine CD11a/CD18 and LktA-induced cytolysis were inhibited by anti-CD11a and anti-CD18 mAbs but not by anti-CD11b and anti-CD11c mAbs in primary bovine alveolar macrophages and neutrophils [281]. To further demonstrate that bovine CD18 is necessary and sufficient for LktA-mediated cytolytic activity, LktA-resistant murine P815 cells were transfected with cDNA encoding bovine CD18 [282]. Transfected cells, stably expressing bovine CD18 paired with murine CD11a, were effectively lysed by LktA and cell lysis was partially inhibited with an anti-bovine CD18 mAb [282]. To demonstrate conclusively that CD11a/CD18 is sufficient to induce cell susceptibility to LktA, bovine CD11a/CD18 was ectopically expressed in the LktA resistant human K562 cells [283]. Subsequent exposure of bovine CD11a/CD18-expressing K562 cells to the toxin induced tyrosine phosphorylation of the cytoplasmic tail of the CD18 subunit, elevation of intracellular calcium ions, and lysis of the cells [283]. In another report, the same group showed that LktA can efficiently bind to the CD18 subunit of both CD11a/CD18 and CD11b/CD18, but the elevation of intracellular calcium ions and phosphorylation of the CD18 tail occurred only when the toxin interacted with the CD11a/CD18 heterodimer [285]. To further identify the exact subunit of bovine CD11a/CD18 used by LktA as a functional receptor, individual monomeric subunits, CD11a or CD18, and heterodimeric CD11a/CD18 were ectopically expressed on the surface of HEK-293 cells [286]. While all three cell variants effectively bound LktA, toxin-induced cytolysis and elevation of intracellular calcium ions were observed only in cells expressing the monomeric CD18 subunit or the heterodimeric CD11a/CD18 integrin [286].

Several different LktA-binding sites on the CD11a/CD18 integrin have been described. Experiments with bovine/murine CD18 chimeras ectopically expressed in P815 cells showed that a region encompassing residues 1–291 of bovine CD18 is crucial for the cytolytic activity of LktA [284]. The coexpression of bovine/human chimeric CD18 molecules with bovine CD11a in K562 cells revealed that a sequence encompassing residues 500–600 of bovine CD18 is required for the binding and biological effects of LktA [289]. This sequence was later narrowed down to residues 541–581 of bovine CD18, which contains the I-EGF-like domain 3 (Figure 7b) [287]. Interestingly, LktA also bound to the CD11a subunit of CD11a/CD18, and the binding could be inhibited by a small molecule inhibitor of the I-domain, the major ligand-binding site of CD11a (Figure 7b) [285]. The inhibition significantly reduced LktA-induced elevation of intracellular calcium ions and tyrosine phosphorylation of the cytoplasmic tail of the CD18 subunit [285]. Later work then showed that the binding site of LktA is formed by residues 5–17 within the signal peptide of ruminant CD18 (Figure 7b), which is not cleaved by the endoplasmic reticulum-resident signal peptidase [288]. The substitution of a single residue in the signal peptide (Q to G at position −5 relative to the cleavage site) resulted in its cleavage and abrogation of LktA-induced cytolysis of cells ectopically expressing cleavable bovine CD18 [288]. In a subsequent proof-of-principle study, a bovine fetus was genetically engineered to express CD18 with the Q(-5)G substitution and leukocytes isolated from this engineered ruminant that expressed CD18 without the signal peptide were completely resistant to LktA-induced cytolysis [290]. All the studies described above indicate that the CD18 subunit of ruminant β_2_ integrins serves as a functional receptor for LktA.

It has further been reported that one of the RTX toxins of *A. pleuropneumoniae*, ApxIIIA, also interacts with the CD18 subunit of the β_2_ integrins [257]. In this work, β_2_ integrin-deficient and ApxIIIA-resistant human K562 cells were used to ectopically express homologous or heterologous CD11a/CD18 heterodimers consisting of porcine, human, or bovine subunits. Only cells expressing the porcine CD18 subunit within the CD11a/CD18 heterodimer were found to be susceptible to ApxIIIA, suggesting that porcine CD18 is necessary to mediate ApxIIIA-induced leukolysis and species-specific toxicity.

CyaA was the first RTX toxin shown to bind to target cells via the β_2_ integrin CD11b/CD18 [53]. The saturable binding of CyaA to various hematopoietic cells correlated with the expression of CD11b/CD18 on the cell surface, and the binding and cytotoxic activities of CyaA were specifically blocked by anti-CD11b but not by anti-CD11a, anti-CD11c, or anti-CD18 mAbs. Moreover, CyaA efficiently bound Chinese hamster ovary (CHO) cells ectopically expressing human CD11b/CD18 but not the cells expressing human CD11a/CD18 or CD11c/CD18 [53,57]. A productive and tight interaction of CyaA with CHO cells expressing CD11b/CD18 required the acylation of the toxin, whereas the N-terminal catalytic AC domain was not necessary for binding of CyaA to the integrin [160]. The major CD11b/CD18 integrin binding site of CyaA was located in the C-terminal RTX domain delimited by residues 1166 to 1281 [160]. Later, we used different glycosidases and an inhibitor of protein N-glycosylation to demonstrate that N-glycosylation of the highly glycosylated receptor CD11b/CD18 is essential for CyaA binding and efficient intoxication of CD11b/CD18-expressing cells [55]. The competitive inhibition of CyaA binding to CD11b/CD18 exclusively by free saccharides, which occur as building blocks of the integrin oligosaccharide complex, then showed that CyaA directly and selectively recognizes the sugar residues of the N-linked oligosaccharide chains of the integrin [55]. In further work, we substituted asparagine residues of the individual N-glycosylation sites of human CD11b and CD18 with glutamine residues that cannot be glycosylated and showed that N-linked oligosaccharide chains in the C-terminal portion of the CD11b subunit are involved in the binding and cytotoxicity of CyaA [291]. Using CHO cells ectopically expressing human CD11b/CD18 variants lacking the α-helical transmembrane segment(s) or containing artificial transmembrane segments, we demonstrated that the integrin transmembrane segments are not directly involved in the invasive AC and pore-forming activities of the toxin [292]. Next, we used human CD11b/CD11c chimeras ectopically expressed together with the human CD18 subunit in CHO cells to show that the binding and cytotoxic activity of CyaA involves interaction with the residues 614–682 of an extracellular domain of CD11b and does not involve interaction with the I-domain, which is the major ligand-binding site of the integrin (Figure 7b) [57]. We proposed that the protein segment containing residues 614–682, and the N-linked oligosaccharide chains of CD11b together form a highly organized structure responsible for the high affinity binding of CyaA to the integrin receptor [291]. Upon CD11b/CD18 binding and insertion into the membrane, the CyaA intermediate permeabilizes cells for influx of extracellular calcium ions, which activate an intracellular Ca^2+^-dependent cysteine protease calpain. Calcium-activated calpain then cleaves the cytoskeletal protein talin, which anchors the integrin to the actin cytoskeleton [293]. This leads to the recruitment of the integrin-CyaA complex into lipid rafts, in which the cholesterol-rich lipid environment promotes the translocation of the AC domain across the cytoplasmic membrane into the cell cytosol [293].

CyaA has been proposed to bind yet another β_2_ integrin on leukocytes, the β₂ integrin CD11a/CD18 [294]. However, the interaction between CyaA and CD11a/CD18 was in stark contrast to the results of Guermonprez and colleagues, who showed that CyaA binds selectively and with high affinity to cells expressing CD11b/CD18, such as macrophages and dendritic cells, while B and T cells expressing only CD11a/CD18 were recognized by the toxin with very low efficacy, like other cells lacking CD11b/CD18 [53]. As further evidence, we expressed equal amounts of human β_2_ integrin molecules on the surface of CyaA-resistant CHO cells and showed that the toxin efficiently bound and intoxicated only the cells expressing CD11b/CD18, whereas it bound and intoxicated CD11a/CD18- or CD11c/CD18-expressing cells with as low efficacy as the CHO cells lacking any β_2_ integrin [57].

Based on the cited literature showing that several RTX toxins specifically interact with the β_2_ integrins on the cell surface, we recently investigated whether the β_2_ integrins could also be the potential receptors for the RTX cytolysin RtxA [262]. We examined the binding and cytotoxicity of RtxA on CHO cells that ectopically expressed three different human β_2_ integrins, CD11a/CD18, CD11b/CD18, or CD11c/CD18. We demonstrated that CHO cells expressing the β_2_ integrins bound similar amounts of RtxA as CHO cells not expressing any β_2_ integrin. Similarly, the viability of β_2_ integrin-expressing CHO cells was decreased after treatment with RtxA in the same time-dependent manner as the viability of CHO cells lacking β_2_ integrins. Analyses of the binding of RtxA to primary mouse macrophages differentiated from bone marrow cells isolated from CD11a knockout (KO), CD11b KO, and control (WT) mice showed that RtxA bound to CD11a KO and CD11b KO macrophages with the same efficacy as to WT macrophages expressing both CD11a/CD18 and CD11b/CD18. Similarly, the viabilities of CD11a KO and CD11b KO macrophages treated with RtxA were reduced in the same time-dependent manner as the viability of the WT macrophages. Thus, the results showed that RtxA, unlike the RTX toxins described above, does not recognize the β_2_ integrins as specific receptors on target cells [262].

### 8.2. β_2_ Integrin Receptor-Independent Interaction

Early reports indicated that some strains of *A. actinomycetemcomitans* have the potential to be beta-hemolytic [295,296,297]. In 2006, Balashova et al. surprisingly demonstrated that the hemolytic phenotype was dependent on the LtxA leukotoxin, which has been repeatedly shown to specifically recognize human and primate leukocytes expressing the β_2_ integrins [61]. Purified LtxA was able to lyse human and sheep erythrocytes, demonstrating that the toxin can also efficiently destroy cells lacking the β_2_ integrins. However, the quantity of LtxA required to lyse erythrocytes was higher than that required to kill leukocytes, indicating the presence of low affinity receptor(s) on erythrocytes [61]. In another report from the same group, the authors investigated how LtxA might recognize the surface of erythrocytes and showed that each of the five different gangliosides (GM1, GM3, GD1a, GD1b, and GT1b), containing at least one sialic acid residue, could completely block LtxA-mediated hemolysis in a dose-dependent manner [275]. In contrast, asialo GM1, which lacks the sialic acid residue, or free sialic acid were unable to completely block hemolysis. This suggested that the sialic acid residue is a necessary component of gangliosides required for the interaction of LtxA with erythrocytes, but is not sufficient on its own to inhibit hemolysis. The results were confirmed in ganglioside-rich C6 rat glioma cells, which are recognized but not killed by LtxA and to which binding of the toxin was successfully blocked by several different gangliosides (GM1, GM3, GD1a and GD3). In contrast, gangliosides could only partially block LtxA-mediated killing of β_2_ integrin-expressing THP-1 cells when the ratio of gangliosides to LtxA was high and the toxin was incubated with THP-1 cells for a short incubation period [275]. From these findings and from previous reports showing that ganglioside-expressing but β_2_ integrin-negative leukocyte cell lines are completely resistant to LtxA-mediated cytotoxicity [48,298,299], the authors concluded that gangliosides act as functional receptors on erythrocytes but not on leukocytes or other cells (e.g., C6 glioma cells) [275]. A later study demonstrated that sialic acid residues are important for LtxA-induced cell lysis, regardless of whether the sialic acid residues are linked to the glycosylated β_2_ integrins or other glycosylated cell surface structures [65]. They found that the preincubation of human or mouse erythrocytes with neuraminidase, an enzyme that catalyzes the hydrolysis of sialic acid residues from various substrates (glycoproteins, glycolipids, and oligosaccharides), significantly decreased LtxA-mediated hemolysis in a concentration-dependent manner. Similarly, the removal of sialic acid residues significantly decreased LtxA-induced lysis of β_2_ integrin expressing K562 cells [65]. These results were consistent with our previous data showing that β_2_ integrin-expressing Jurkat T cells pretreated with a mixture of neuraminidase and two other glycosidases, PNGase F and Endo H, were less sensitive to LtxA than untreated Jurkat T cells [55]. In summary, LtxA can interact with the surface of β_2_ integrin-negative cells via negatively charged sialic acid residues that are part of numerous glycosylated cell surface structures such as glycoproteins, glycolipids, and even gangliosides.

Interestingly, the interaction of LtxA with both CD11a/CD18-negative and CD11a/CD18-positive cells was shown to increase cytosolic Ca^2+^ levels, suggesting that the event is independent of the interaction between LtxA and the integrin [300]. However, only CD11a/CD18-expressing cells were killed by the toxin. It was suggested that LtxA initially binds to both toxin-sensitive and toxin-resistant cells by passive adsorption [300], most likely via negatively charged sialic acid residues, as described above. Increased levels of cytosolic Ca^2+^ in CD11a/CD18-positive cells subsequently activate the protease calpain that cleaves talin, a protein that anchors CD11a/CD18 to the cytoskeleton. This results in mobilization to and subsequent clustering of CD11a/CD18 and LtxA in cholesterol- and sphingolipid-rich membrane rafts. The association of CD11a/CD18 and LtxA within lipid rafts is crucial for LtxA-mediated cytotoxicity, as only cells expressing CD11a/CD18 were killed by the toxin. Moreover, cholesterol depletion experiments showed that raft integrity is necessary for the function of LtxA [300]. Later, surface plasmon resonance and differential scanning calorimetry measurements showed that LtxA binds to phospholipid bilayers containing 40% cholesterol with 4 orders of magnitude higher efficacy than to phospholipid bilayers lacking cholesterol [63]. Primary sequence analysis revealed that LtxA contains two so-called cholesterol recognition/interaction amino acid consensus (CRAC) motifs with the pattern L/V-(X)(1–5)-Y-(X)(1–5)-R/K (where (X)(1–5) represents one to five residues of any amino acid) [276]. Peptides corresponding to both motifs bound cholesterol, but only the peptide corresponding to the CRAC site between residues 333–339 competitively inhibited the binding of LtxA to this sterol and the ability of the toxin to kill Jurkat cells. Moreover, mutations in this CRAC motif abolished the ability of LtxA to kill Jurkat cells [63]. Later, the same group showed that the removal of cholesterol from the membrane with methyl-β-cyclodextrin or mutation of the CRAC motif of LtxA blocks the activity of the toxin in THP-1 cells [277]. Thus, all these results show that LtxA uses a specific cholesterol-binding motif for membrane association.

Using different approaches, Cortajarena et al. demonstrated that HlyA recognizes glycophorin as a receptor on the surface of human erythrocytes [264]. Preincubation of HlyA with increasing concentrations of purified horse glycophorin reduced the hemolytic activity of the toxin on horse erythrocytes, an anti-glycophorin antibody significantly reduced HlyA-mediated lysis of erythrocytes, and immobilized HlyA bound glycophorin from a detergent lysate of erythrocyte ghosts. In addition, glycophorin-containing liposomes were more sensitive to HlyA than pure liposomes, and this sensitivity was reversed when glycophorin-containing liposomes were treated with trypsin [264]. The same group later reported that HlyA with a deletion of residues 914–936 exhibits a 10,000-fold reduction in hemolytic activity compared with intact HlyA [265]. The HlyA mutant was unable to bind erythrocytes or pure glycophorin in an affinity column. Moreover, the HlyA-derived peptide (W914–R936) bound glycophorin reconstituted in liposomes and protected erythrocytes from hemolysis induced by intact HlyA. All these results demonstrated that glycophorin acts as a receptor for HlyA and is recognized by residues 914–936 of the toxin, which form a major glycophorin-binding site [264,265]. Interestingly, glycophorin is not a receptor for LtxA [275], although LtxA can lyse erythrocytes isolated from various species [65]. Experimental data are needed to reveal whether glycophorin is recognized as a receptor by RtxA.

In 2009, Herlax et al. showed that cholesterol-depleted erythrocytes are less sensitive to the hemolytic activity of HlyA than control erythrocytes and that HlyA is associated with detergent-resistant erythrocyte membranes enriched in cholesterol and sphingomyelin [162]. Later, different biochemical and biophysical assays were used to demonstrate the direct interaction of HlyA with cholesterol but not with sphingomyelin [266]. Moreover, 20 potential cholesterol binding motifs (7 CRAC motifs and 13 inverted CRAC motifs, so-called CARC motifs with the consensus pattern R/K-(X)(1–5)-Y/F-(X)(1–5)-L/V) [301]) were identified within the HlyA sequence. It has been suggested that the interaction of HlyA with cholesterol favors a conformational state of the toxin that allows proper membrane insertion and pore formation [266].

The first experiments to study the interaction of CyaA with the surface of eukaryotic cells were performed at a time when the CD11b/CD18 integrin was not yet known to be a specific receptor for CyaA. Gable and coworkers showed that the cytotoxicity of CyaA to polymorphonuclear leukocytes could be inhibited by pretreating the cells with neuraminidase or by preincubation of the toxin with bovine brain gangliosides [270]. A later study then showed that preincubation of CyaA with different types of gangliosides (GM1, GM3, and GT1b) inhibits the CyaA-catalyzed cAMP intoxication of CHO cells lacking CD11b/CD18 [271]. Later work showed that the pretreatment of GM1-positive and CD11b/CD18-negative human erythrocytes and K562 cells with GM1-binding cholera toxin subunit B (CTB) decreased CyaA binding by ~30% [272]. It indicated that CTB competes with CyaA for a binding site on GM1, which is likely formed by terminal galactose and sialic acid residues in GM1 [272]. These results are consistent with our previous data showing that an initial interaction of CyaA with CD11b/CD18-expressing cells depends on selective recognition of N-linked oligosaccharide chains of CD11b/CD18 by the toxin [55]. Indeed, the binding of CyaA to CHO-CD11b/CD18, J774A.1, and human neutrophils was decreased by ~80% when terminal sialic acid residues of CD11b/CD18 and other cell surface glycoproteins were removed by neuraminidase. An almost complete loss of CyaA binding to cells was observed when N-linked oligosaccharides of surface glycoproteins were removed by the glycosidase PNGase F or when the N-glycosylation of newly synthesized proteins was blocked with the nucleoside antibiotic tunicamycin. Moreover, the binding of CyaA to cells was inhibited by 40% to 80% in the presence of free saccharides (e.g., sialic acid, N-acetyllactosamine, D-mannose, etc.), which occur as building units of the N-linked oligosaccharide chains of CD11b/CD18, but not in the presence of free saccharides that are not part of the integrin oligosaccharide chains [55]. All these results indicate that sialic acid residues together with specific saccharides can be recognized by CyaA not only on the glycosylated CD11b/CD18 integrin but also on glycosylated cell surface structures present on CD11b/CD18-negative cells.

In 2004, Martin et al. reported that cholesterol substantially increases the rate of CyaA-induced membrane lysis, measured as efflux of fluorescent liposomal content, in a dose-dependent manner [267]. Later, CyaA binding to erythrocytes was shown to be reduced when cells were preincubated with methyl-β-cyclodextrin, suggesting that membrane microdomains rich in cholesterol and sphingomyelin may be involved in the binding of the toxin to erythrocyte membranes [268]. Indeed, CyaA bound with a significantly higher efficiency to liposomes formed from sphingomyelin than to liposomes made from pure phosphatidylcholine, and the toxin binding was even enhanced when liposomes were made from a mixture of sphingomyelin and cholesterol [268]. Interestingly, we showed that the predicted cholesterol binding sites (five CRAC motifs) within the CyaA molecule were not involved in binding the toxin to erythrocytes or to CD11b/CD18-positive J774A.1 cells, and the binding of CyaA to the cells was not inhibited by free cholesterol [139]. Recent results suggest that cholesterol increases the lytic potency of CyaA by favoring its membrane insertion and oligomerization, steps that are necessary for the toxin to accomplish membrane permeabilization and cell lysis [269].

Recently, we investigated whether the binding and cytotoxic activities of RtxA also depend on the recognition of glycosylated membrane structures, as previously shown for several other RTX toxins [262]. We showed that the binding and cytotoxic activity of RtxA could be significantly decreased when different cell types were preincubated with neuraminidase. Moreover, the binding of RtxA to the cells was also partially blocked by free sialic acid. It suggested that peripheral sialic acid residues of cell surface components such as glycoproteins or gangliosides are important for both the binding and cytotoxicity of RtxA. We proposed that the interaction between RtxA and target cells is mediated by the positively charged lysine and arginine residues of the RTX domain of RtxA and the peripheral, negatively charged sialic acid residues linked to cell surface structures. Furthermore, we demonstrated that both enzymatic (PNGase F, O-glycosidase) and inhibitor-mediated (tunicamycin, benzyl-2-acetamido-2-deoxy-α-D-galactopyranoside) removal of N- or O-linked oligosaccharide chains from cell surface glycosylated structures resulted in a significant loss of RtxA binding, and deglycosylated cells were more resistant to the cytotoxic effect of RtxA than untreated cells. Thus, RtxA not only recognizes the sialic acid residues but also other saccharide units of the cell surface glycoproteins on the surface of target cells [262].

Using different biochemical and biophysical methods, we demonstrated that membrane cholesterol is important for the binding and cytotoxic activity of RtxA [37]. A strong binding of fluorescently labeled RtxA to giant unilamellar vesicles (GUVs) composed of 75% 1-palmitoyl-2-oleoyl-*sn*-glycero-3-phosphocholine (POPC) and 25% cholesterol was observed, whereas the binding of the toxin to GUVs composed of 100% POPC was rather weak. Surface plasmon resonance measurements then showed a stronger affinity of RtxA for the cholesterol-containing POPC membrane than for the pure POPC membrane. Moreover, RtxA bound with 2–3-fold higher efficacy to the wells of an ELISA plate coated with cholesterol-BSA than to the wells coated with free BSA, showing that the toxin is able to interact with cholesterol independently of the presence of other membrane components. Moreover, erythrocytes preincubated with methyl-β-cyclodextrin bound significantly lower amounts of RtxA than untreated erythrocytes. Correspondingly, RtxA preincubated with free cholesterol exhibited significantly reduced capacity to lyse erythrocytes. Using primary sequence analysis, we found that RtxA contains five potential cholesterol binding motifs (two CRAC and three CARC motifs) located adjacent to or within the predicted pore-forming domain of the toxin. Substitutions of the key tyrosine residues within the CRAC–CARC motifs of RtxA then indicated that the tandem CARC–CRAC motifs between residues 340 and 354 may be responsible for the interaction of the toxin with membrane cholesterol [37]. All these results showed that glycosylated structures on the cell surface and membrane cholesterol together act as the major membrane components used by RtxA to interact with target cells. Since glycosylated structures are present on the surface of all mammalian cells and cholesterol is a key structural component of all animal membranes, our results explain the previously observed promiscuity of RtxA binding to a wide spectrum of cells from various species and indicated that RtxA can be classified as a broadly cytolytic RTX hemolysin [37,262].

### 8.3. Interaction via Outer Membrane Vesicles

Commensal and pathogenic Gram-negative bacteria produce outer membrane vesicles (OMVs) during their normal growth [302,303,304,305,306,307]. OMVs are spherical, bilayered nanostructures that range in size from 20 to 400 nm. OMVs are formed by the blebbing of the bacterial outer membrane (OM), so their layer composition is very similar to that of OM and mainly includes phospholipids, lipopolysaccharides, lipooligosaccharides, and proteins found in OM. The interior of OMVs contains many parts of the parent bacteria such as DNA, RNA, peptidoglycan, enzymes, and other proteins. These components are called microorganism-associated molecular patterns (MAMPs), through which OMVs interact with host cell pattern recognition receptors (PRRs). This interaction leads to the release of cytokines, chemokines, and antimicrobial peptides, so that OMVs can trigger both proinflammatory and anti-inflammatory responses in the host organism. In addition, OMVs can contain virulence factors, making them important players in bacterial pathogenesis [302,303,304,305,306,307]. For example, RTX cytotoxins such as LtxA, HlyA, and CyaA have been shown to be associated with OMVs [308,309,310,311,312].

In 2011, Maldonado and colleagues identified for the first time OMVs produced by nine different strains of *K. kingae* [39]. Transmission electron micrographs showed that the size of the OMVs ranged from 50 to 200 nm in diameter. To quantify the production of OMVs, they purified OMVs produced by nine *K. kingae* strains growing under the same conditions and compared their amounts. Based on the results, they divided the *K. kingae* strains into two categories: strains with high and low production of OMVs. Interestingly, all *K. kingae* joint isolates belonged to the group with high OMV production, suggesting that the production of OMVs may be beneficial for the bacterium in causing septic arthritis. To further investigate OMV properties, they selected *K. kingae* strain PYKK081 with high OMV production isolated from a patient with septic arthritis. The PYKK081 OMVs contained two virulence factors, PilC2 pilus adhesin and especially the RtxA toxin, which made them cytotoxic to various human and mouse cells, such as monocytes, T and B lymphoblasts, and megakaryoblasts. However, the OMV fraction had an insignificant hemolytic effect on human and sheep erythrocytes, of which only 30% were lysed after 16 h of incubation with OMVs [39].

**Figure 8:**
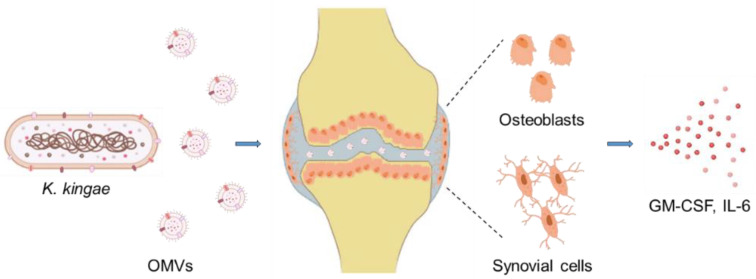
The proposed mechanism of action of *K. kingae* strain PYKK081 OMVs in human bone. OMVs released by *K. kingae* are internalized by human osteoblasts and synovial cells. This leads to increased production of two cytokines, GM-CSF and IL-6, which might be involved in the signaling response of infected bone and joint tissues during *K. kingae* infection. This image was created using BioRender.com.

*K. kingae* primarily causes osteoarticular infections, mainly affecting bone and joint tissues [3,89]. Therefore, the effect of OMVs on human osteoblasts and synovial cells was investigated [39]. Interestingly, the cytotoxic effect of PYKK081 OMVs on human osteoblasts was not demonstrated. However, purified PYKK081 OMVs were rapidly taken up by human osteoblasts (hFOB 1.19) and synovial cells (SW 982). Flow cytometry results showed that the first internalization of FITC-labeled OMVs occurred within 15 min after addition of the labeled OMVs to hFOB 1.19 and SW 982 cells. Moreover, after two hours, almost all labeled OMVs were internalized by these target cells. Maldonado et al. also investigated the possible proinflammatory effect of PYKK081 OMVs on hFOB 1.19 and SW 982 cells. They examined the changes in the production of 20 inflammatory cytokines by immunoassay analysis. The hFOB 1.19 and SW 982 cells showed increased production of 2 cytokines 24 h after treatment with OMVs. Human granulocyte-macrophage colony-stimulating factor (GM-CSF) and IL-6 levels were increased by ~6.1-fold and ~2.7-fold, respectively, compared with negative control (Figure 8) [39]. However, it is not yet clear whether the increased levels of these cytokines were caused by membrane components of the OMVs or the RtxA toxin.

## 9. Formation of Membrane Pores by RtxA and Other RTX Toxins

The first report on the formation of transmembrane pores by RTX toxins was published in 1983 by Jorgensen et al. [31]. To explain the observed calcium accumulation and potassium depletion in erythrocytes after treatment with HlyA, the authors proposed that the toxin could generate a cation-specific membrane pore [31]. Three years later, Bhakdi et al. performed a set of straightforward experiments showing that HlyA can damage cell membranes by insertion into the lipid bilayer and the formation of a hydrophilic transmembrane pore [32]. Further studies confirmed these initial reports and also showed that the N-terminal pore-forming hydrophobic domain of the RTX toxins is essential for their pore-forming (hemolytic) activity [34,35,36,127,131,133,134,135,136], as we have described in detail in a chapter above.

**Figure 9:**
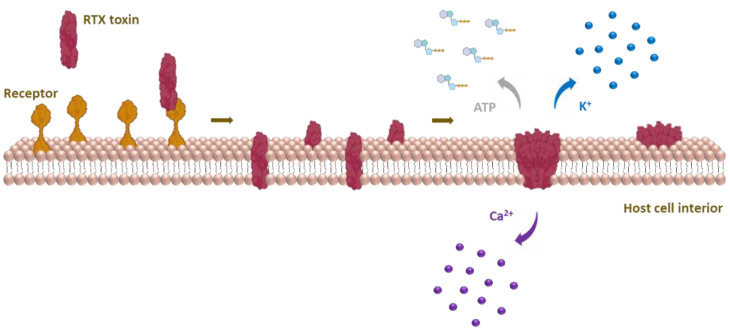
The pore-forming activity of RTX toxins. Although the exact mechanism of oligomerization and pore-forming activity of RTX toxins in vivo is still largely unknown, an approximate mechanism can be given. The RTX toxin interacts with a host receptor and inserts into the membrane in a monomeric form. The monomers interact with each other to form an oligomeric complex. The number of monomers that form an oligomeric structure and the mechanism of formation are still unknown. The oligomeric complex acts as a membrane pore through which calcium ions flow into the cell cytoplasm and potassium ions flow out of the cell. HlyA and LtxA also enable ATP release from host cells directly through the membrane pores formed by the toxins. This image was created using BioRender.com.

Based on current knowledge, it is generally accepted that the pore-forming activity of RTX toxins requires the oligomerization of two or more toxin molecules in the membrane of target cells (Figure 9) [34,35,149,162,203,313,314]. Despite the fact that the study of the oligomerization of RTX toxins is complicated by their natural tendency to form inactive aggregates in solution [51], the following pieces of evidence show that RTX toxins form oligomeric pores in the membrane [164]:

1. The steep dependence of membrane conductance on RTX toxin concentration in the BLM system, which can be explained by the assumption that there is an association-dissociation equilibrium between nonconducting monomers and conducting oligomers [34,133,313].
2. Complementation analysis of inactive mutants of CyaA [315] and HlyA [316] resulted in partial recovery of their hemolytic activities. The combination of truncated, non-overlapping CyaA and HlyA variants restored the ability of the toxins to permeabilize the cell membrane [315,316]. Experiments with truncated variants of CyaA suggested that functional complementation might occur via calcium-binding nonapeptide repeats in the C-terminal part of the toxin molecule [191].
3. The acylation status of CyaA appears to modulate the propensity of the toxin to form oligomeric membrane pores, as hemolytic (pore-forming) activity was influenced by the attachment of various fatty acyl chains [133,148,149,150]. It was also suggested that the acyl chains in HlyA promote the protein–protein interactions necessary for oligomerization of the toxin [162].
4. FRET analysis revealed selective self-association of CyaA molecules in solution leading to oligomeric complexes [314].
5. The strongest evidence for oligomerization of RTX toxins was provided in 2009 when FRET analysis of HlyA revealed its oligomerization in membranes of sheep erythrocytes [162] and oligomeric complexes of CyaA formed in erythrocyte membranes were detected by immunogold labeling and blue native polyacrylamide gel electrophoresis [203].

Although the pores formed by RTX toxins are irreversibly anchored in the eukaryotic membrane, they are unstable, and their formation and decay appear to be very dynamic processes that depend on factors such as membrane composition and fluidity, toxin concentration, temperature, time, or toxin acylation [36,129,162,317,318]. The pore-forming properties of RtxA and three other RTX toxins are listed in Table 4 and described in detail in the following text.

Experiments in planar lipid bilayers showed that the pores formed by LtxA exhibit a complex pattern with multiple conductance states of 118, 262, and 406 pS in solutions with 140 mM NaCl, with the first two states showing voltage-dependent pore gating [319]. Tests with osmotic protectants in LtxA-sensitive HL-60 cells indicated that the functional diameter of the pores formed by the toxin is ~0.9 nm [320]. Direct electrophysiological evidence for the pore-forming activity of LtxA in HL-60 cells was obtained using patch electrode recordings of whole-cell currents [321]. Subsequent studies examining the interaction of LtxA with liposomes using various biophysical methods suggested that the toxin mediates membrane damage by destabilizing the membrane rather than by forming a transmembrane pore [322].

**Table 4:** Properties of pores formed by RTX toxins.

RTX Toxin	Pore Diameter (nm)	Single-Pore Conductance (pS) ^1^	Single-Pore Lifetime (s)	Ref.
**RtxA**	~1.9	~400, ~419 *	~0.24 *, 2.12 *	[36,37]
**HlyA**	~1–3	~500	~2	[32,33,34]
**CyaA**	~0.6–0.8	~9–11	~2	[133,139,148,196,323,324]
**LtxA**	~0.9	~406, 262, 118	NA ^2^	[141,319,320]

^1^ Single-pore conductance values of RTX toxins were determined in 100–150 mM KCl (exact values are given in the text). The asterisk indicates the most frequent values determined with recombinant RtxA. ^2^ NA: no data available.

As mentioned earlier, one of the first reports of the pore-forming activity of HlyA was published by Bhakdi et al. [32]. The authors measured K^+^ efflux and radioactive marker ^45^Ca^2+^ influx using rabbit erythrocytes and suggested that HlyA can damage cell membranes by forming a hydrophilic transmembrane pore with an effective diameter of ~3 nm. Since the HlyA molecule was isolated from deoxycholate-solubilized erythrocyte membranes by sucrose density gradient centrifugation exclusively in a monomeric form, it was suggested that pore formation by HlyA might be caused by the insertion of toxin monomers into the target membrane. However, it was also discussed that it cannot be ruled out that the pores of HlyA consist of toxin oligomers dissociated by deoxycholate [32]. Shortly thereafter, the same group reported that HlyA forms a voltage-dependent, cation-selective, and ion-permeable pore with a diameter of ~2 nm in a planar bilayer membrane [33]. Since it was observed that the total number of bound HlyA molecules is linearly proportional to the concentration of HlyA in solution, indicating single-hit kinetics of the lytic process, it was suggested that the toxin is active as a monomer [33]. In another report, Menestrina supported the single-hit mechanism of pore formation by HlyA by additional experiments [325]. He prepared small unilamellar lipid vesicles (SUVs) loaded with the fluorescent agent calcein and measured its release in the presence of HlyA and a pore-forming control protein, the α-toxin of *S. aureus*, which is known to form heptameric toxin complexes in cell membranes. While the dependence of the permeabilization of SUVs on the concentration of HlyA was linear, the kinetic of the response of the α-toxin was multiexponential. This suggested that HlyA is active in a monomeric form [325]. Later experiments showed that the membrane activity of HlyA is rather low in membranes made of pure lipids (e.g., phosphatidylcholine or phosphatidylserine) but can be increased by many orders of magnitude in membranes made of asolectin [34]. Single-channel recordings and zero-current membrane potential experiments revealed that the membrane activity of HlyA was increased due to the formation of cation-selective, ion-permeable pores with a single-pore conductance of ~500 pS in 150 mM KCl and an effective pore diameter with a lower limit of ~1.0 nm. The increase in HlyA conductance in a steep concentration-dependent manner suggested that several HlyA molecules might be involved in the formation of a conducting unit, which exhibited an association-dissociation reaction with a mean lifetime of ~2 s at 20 mV [34]. This conjecture was supported by experiments showing that HlyA lysed large unilamellar vesicles loaded with fluorescent solutes with a second-order kinetic behavior, implying that HlyA acted as a dimer [326]. Later, the patch-clamp technique was used to show that HlyA forms pores in the plasma membrane of human macrophages similar to those in planar lipid membranes [327].

Initial osmotic protection experiments performed with CyaA on erythrocytes suggested that it forms a membrane pore less than 0.62 nm in diameter, with a size 3–5 times smaller than that of HlyA [323]. This fits well with the fact that the pore-forming (hemolytic) activity of CyaA is not as important for *B. pertussis* virulence as the ability of the toxin to intoxicate cells by cAMP [328,329]. CyaA also produced cation-selective, ion-permeable pores in phospholipid bilayers that were strongly influenced by the polarity and magnitude of the membrane potential, and whose membrane conductance was dependent on calcium ions [313]. In addition, CyaA variants with no detectable cell-invasive AC and hemolytic activity exhibited low or no conductance. Measurements of the concentration dependence of hemolytic activity and transmembrane conductance in the phospholipid bilayer indicated that an oligomer is involved in pore formation by CyaA and suggested that three or more toxin molecules are involved in the oligomerization process [313]. In the same year, CyaA was reported to form small cation-selective membrane pores with a diameter of 0.6–0.8 nm using the lipid bilayer assay [133]. Similar to HlyA [34], the increase in membrane conductance after the addition of CyaA to membranes made of pure lipids was rather small compared to membranes made of lipid mixtures such as asolectin. CyaA formed rather small transient ion-permeable pores in asolectin membranes with a single-pore conductance of 27 pS in 1 M KCl, which was much lower than that of HlyA under identical conditions (1500 pS) [133]. In 150 mM KCl, the single-pore units of CyaA in asolectin membranes exhibited a conductance ranging from 9 to 11 pS [139,148,196]. Furthermore, experiments with CyaA variants showed that both CyaC-mediated acylation of CyaA and the pore-forming domain of the toxin, but not the AC and RTX domains, are required for pore formation in bilayer membranes [133]. Additional experiments with CyaA in lipid bilayer membranes showed that pore properties, such as pore lifetime and size, depend on the orientation of the electrical potential across the membranes [324]. When the voltage on the cis side (the side of addition of the toxin) was positive, CyaA formed regular pores with a single-pore conductance of ~45 pS in 1 M KCl and a lifetime of ~2 s. The CyaA pores were formed by CyaA oligomers, and their voltage dependence was enhanced by calcium ions. However, when the cis side was set to a negative potential, the toxin pores were not well defined and exhibited reduced pore-forming activity and a very short lifetime [324]. Our previous results suggested that the negative charges of the aspartate and glutamate residues located within the AC-to-Hly-linking segment of the CyaA molecule play an important role in restriction of the size of toxin pores and in control of the frequency of pore formation [196]. It indicated that the AC-to-Hly-linking segment is responsible for the smaller size and low cell-permeabilizing capacity of CyaA pores compared to the membrane pores of typical RTX hemolysins [196]. While the formation of oligomeric pores of CyaA in the target membrane is required for the pore-forming (hemolytic) activity of the toxin [35,134,149,203,313], it was shown that oligomerization of the toxin is not a prerequisite for the interaction of CyaA with the cell membrane [35,330,331]. It appears that the monomeric form of CyaA is incorporated into the membrane and is sufficient to trigger the fluxes of potassium and calcium ions and to deliver the AC domain into the cell interior [35,149,203,332].

In 2015, using planar lipid bilayers made of asolectin, Uribarri et al. showed that RtxA purified from *K. kingae* strain PYKK081 forms pores with a single-pore conductance of ~400 pS in 100 mM KCl and an apparent diameter of ~1.9 nm [36]. The membrane pores formed by RtxA were cation-selective and showed strong voltage-dependent gating. The reported size of the RtxA pores was within the range of pore sizes observed for many other RTX toxins. Nevertheless, partial differences in single-pore conductance, ion selectivity, and pore diameter may reflect some variations in the pore structures of RtxA and other RTX toxins [36]. Later, we used planar lipid bilayers made of asolectin to characterize the pore properties of recombinant RtxA and unacylated proRtxA purified from an *E. coli* expression system [37]. We showed that RtxA forms membrane pores with three well-distinguishable conductance states in 150 mM KCl, with the most frequent values of ~419 pS (80% frequency of occurrence), ~210 pS (10% frequency of occurrence), and ~38 pS (10% frequency of occurrence). The majority of RtxA pores had a most frequent shorter lifetime of ~0.24 s, while the remaining RtxA pores had a most frequent longer lifetime of ~2.12 s and a minor proportion of toxin pores were opened for tens of seconds. The membrane pores formed by proRtxA had similar single-pore conductance states (~433, ~227, and ~44 pS) and the most frequent shorter lifetime (0.23 s) as RtxA. The longer most frequent lifetime of proRtxA was ~2 times higher than that of RtxA (4.11 s and 2.12 s, respectively). Nevertheless, RtxA and proRtxA differed significantly in their overall membrane activity (~8-fold), suggesting that unacylated proRtxA was impaired in its proper insertion into the membrane and/or in its tendency to form oligomeric membrane pores with the same capacity as the acylated RtxA toxin [37].

Although RTX toxins have been thoroughly studied in the last decades, more data need to be obtained to reveal the precise molecular mechanisms of pore formation and to describe the 3D structures of the RTX pores embedded in the membrane.

## 10. Effects of RtxA and Other RTX Toxins on Host Cells

The biological effects of RTX toxins on host cells depend mainly on toxin concentration, duration of exposure, and cell type [119,327]. At very low, sublytic toxin concentrations, which may occur at greater distances from the site of bacterial infection, RTX toxins may trigger multiple pathological effects, most likely preferentially in susceptible cells expressing high affinity receptors, such as the leukocyte-restricted β_2_ integrins described above [48,53,257,258,327]. Pathological effects may include the inhibition of phagocytosis, the stimulation or suppression of cytokine and inflammatory lipid mediator release, the modulation of signaling and proteolytic cascades, or the induction of cell cycle arrest and cell apoptosis [43,333,334,335,336,337,338,339,340,341,342,343,344,345,346]. These effects may modulate host cell physiology and survival and may or may not depend on the pore-forming activity of RTX toxins, as direct interaction of the toxins with cell surface receptors may trigger corresponding intracellular signaling pathways [283,285,327,347]. At higher toxin concentrations, such as those found in the vicinity of the bacteria, the number of membrane pores formed in target cells may be relatively large, leading to destabilization of the lipid bilayer and cytoskeleton, as well as osmotic imbalance, causing uncontrolled water influx with subsequent cell swelling and colloid-osmotic lysis [31,32,35,320,327]. These processes can, for example, suppress the ability of immune cells to kill invading bacteria or result in extensive damage to the underlying epithelium, facilitating the penetration of bacteria into the host tissue [58,59,327,348,349]. At higher concentrations, RTX toxins are likely to utilize low affinity receptors that are widely distributed on the surface of target cells and are less specific [61,65,262,271,272,275,327].

Several biological effects of LtxA that may be involved in the pathogenesis of localized aggressive periodontitis caused by *A. actinomycetemcomitans* have been previously described. Experiments with the B-cell hybridoma cell line HS-72 showed that LtxA induces apoptosis by a Bcl-2-inhibitable mechanism and cell cycle arrest in the G2/M phase [338]. Similarly, LtxA irreversibly inhibited cell proliferation through a cell cycle arrest in the G2/M phase in human microvascular endothelial cells hCMEC/D3 [345]. In addition, LtxA induced caspase-dependent apoptosis in hCMEC/D3 cells and increased the expression of the adhesion molecules ICAM-1 and VCAM-1. It was suggested that the decreased cell viability and apoptotic death of microvascular endothelial cells induced by LtxA might lead to the degeneration of microvasculature and consequent destruction of gingival tissue [345]. Other studies demonstrated that LtxA induces the release of proteolytic enzymes from the granules of human polymorphonuclear leukocytes, including active matrix metalloproteinase 8, which degrades type I collagen [339,350]. It was suggested that the LtxA-induced release of proteolytic enzymes from infiltrated cells may contribute to the progression of periodontitis [339,350]. LtxA also triggered the abundant production and secretion of pro-inflammatory IL-1β by human macrophages, which are found in tissues from periodontal lesions [351,352]. This suggested that the secretion of pro-inflammatory cytokines into surrounding tissues could cause an imbalance in the host inflammatory response and stimulate pathogenic cellular mechanisms [351,352]. A recent study showed that the LtxA-mediated lysis of human neutrophils resulted in the release of neutrophil elastase, which caused the detachment and death of human gingival epithelial cells and fibroblasts [353]. This suggested that the LtxA-mediated release of neutrophil elastase could cause the pathophysiological breakdown of periodontal tissue and thereby exacerbate periodontitis [353].

Several studies have described pathophysiologically significant events in host cells treated with purified HlyA or HlyA-producing *E. coli* strains. In 1989, Bhakdi et al. reported that HlyA is a very potent leukocidin that causes membrane permeability defects in human polymorphonuclear neutrophils at a very low concentration of ~1 ng/mL, resulting in the efflux of intracellular ATP and influx of propidium iodide [43]. Membrane permeabilization was a rapid process, accompanied by exocytosis of granules and inhibition of phagocytic killing capacity of the cells [43]. One year later, the same group showed that concentrations of 250–2000 ng/mL of purified HlyA caused an irreversible and rapid decrease in intracellular ATP in human monocytes to levels below 20% of control values within 60 min [334]. Moreover, subcytotoxic doses (10–200 ng/mL) of HlyA triggered rapid release (60–120 min) of high levels of IL-1β from monocytes, but failed to induce the production of higher levels of tumor necrosis factor-α (TNF-α). Similarly, the infection of monocytes with HlyA-producing *E. coli* at a MOI of 0.3–3 but not with the HlyA-lacking *E. coli* variant resulted in a ~50% depletion of total intracellular ATP within 90 min and stimulation of IL-1β release but not TNF-α [334]. Additional experiments with HlyA-positive and HlyA-negative *E. coli* bacteria showed that non-toxic concentrations of HlyA did not stimulate the release of TNF-α, IL-6, and IL-1β from peripheral human monocyte, lymphocyte, and basophil cell suspension [336]. In a later work, two isogenic *E. coli* strains that did or did not produce HlyA were injected into mice, and the sera were assayed for TNF-α and IL-1α [354]. While the HlyA-deficient *E. coli* strain caused no mortality and no significant elevation of serum TNF-α or IL-1α levels, the HlyA-positive strain caused significant mortality and elevation of serum IL-1α levels but no significant elevation of TNF-α levels [354]. It demonstrated that HlyA induced a cytokine response in vivo that was similar to that previously showed in vitro by Bhakdi et al. [334,354]. Experiments with *E. coli* strains producing acylated HlyA or unacylated proHlyA then demonstrated that the bacterium has to produce acylated HlyA to cause hemolysis, increase its virulence in vivo, and trigger IL-1β release from monocytes in vitro [355]. Further work with a virulent isolate of *E. coli* demonstrated that HlyA is critical for triggering both cell death and NLRP3 inflammasome-mediated IL-1β maturation and release in human macrophages [356]. This was recently confirmed with purified HlyA, which, in contrast to proHlyA, induced the activation of the NLRP3 inflammasome in a potassium-dependent manner and activation of caspase-1, leading to IL-1β maturation and its release from THP-1 derived macrophages [357]. Moreover, potassium efflux triggered by HlyA resulted in mitochondrial dysfunction and cell death [357]. All these observations suggest that the HlyA toxin may efficiently impair immune cells and thereby play an important role in the severity of uropathogenic *E. coli* infections.

Experiments in primary rat renal epithelial cells suggested that HlyA-containing culture supernatants stimulated constant, low-frequency oscillations in intracellular calcium ions that depended on calcium influx through voltage-operated L-type calcium channels and from internal stores controlled by inositol triphosphate [358]. HlyA-induced calcium oscillations also stimulated the production of the pro-inflammatory cytokines IL-6 and IL-8, frequently found at elevated concentrations in urine and serum of patients who suffer from acute pyelonephritis [358]. However, later work revealed that HlyA-induced oscillations of intracellular calcium ions were not due to deregulation of physiological calcium ion channels, but derived from pulsed influxes of calcium ions as a consequence of the formation and rapid closure of HlyA pores in cell membranes [359].

Using 5637 human bladder epithelial cells, Wiles et al. showed that HlyA action effectively inhibits the activation of Akt (also known as protein kinase B, PKB), an important regulator of host cell metabolism, proliferation, survival, and inflammatory responses [340]. HlyA abrogated Akt activation via an extracellular calcium-dependent, potassium-independent process that required insertion of HlyA into the host cell membrane and subsequent formation of a toxin pore. Studies with inhibitors indicated that the inactivation of Akt by HlyA is accompanied by the abnormal stimulation of host protein phosphatases such as protein phosphatase-2B (PP2B), which is regulated by calcium ions and can dephosphorylate Akt [340]. Similar to other PFTs (*S. aureus* α-toxin, *V. cholerae* cytolysin, and streptolysin O), HlyA triggered mitogen-activated protein kinase (MAPK) p38 activation in the human keratinocyte line HaCaT by causing a loss of cellular potassium ions [341]. Interestingly, MAPK p38 was shown to be a survival protein in cells treated with bacterial PFTs [360].

In 2009, Skals et al. showed that the ATP scavenger apyrase inhibited the HlyA-induced hemolysis of human, murine, and equine erythrocytes, suggesting that extracellular ATP is required for HlyA-induced hemolysis [361]. Experiments with different inhibitors then showed that the pores formed by HlyA in the erythrocyte membrane trigger the activation of ATP-gated purinergic receptors P2X_1_ and P2X_7_ (membrane cation channels) to mediate the full hemolytic effect. Moreover, non-selective inhibitors of the transmembrane channel pannexin 1, which allows the release of ATP and interacts with the P2X receptors, reduced hemolysis in HlyA-treated erythrocytes. This suggested that the activation of P2X receptors and pannexin channels enhances hemolysis induced by HlyA [361]. Later, the same group showed that LtxA also causes P2X receptor-dependent lysis of human erythrocytes [362]. Similarly, we showed that the hemolytic potency of ApxIA, which forms pores of ~2.4 nm in diameter, is also reduced in the presence of an ATP scavenger or P2X_7_ receptor antagonists [56]. However, the antagonists of purinergic signaling had no effect on the hemolytic potency of CyaA, which forms narrower pores of ~0.6–0.8 nm in diameter. Nevertheless, when the CyaA pore size and propensity of CyaA to form pores were increased by the deletion of residues 6–489, including the AC domain and an adjacent segment, a P2X_7_ receptor antagonist inhibited the enhanced hemolytic activity of the larger pores formed by the CyaA variant. This suggested that the size of membrane pores formed by RTX toxins plays an important role in purinergic amplification of cell lysis [56]. Interestingly, both HlyA and LtxA allowed acute ATP release from human erythrocytes and artificial membranes directly through the membrane pore formed by the toxins [141]. In addition, the inhibition of P2X receptors reduced the damage of human monocytes induced by HlyA and LtxA [363]. A recent study then demonstrated that LtxA-treated Jurkat lymphocytes release ATP via pannexin 1 channels and the released ATP activates the P2X_7_ receptor, resulting in the mobilization of intracellular calcium ions, activation of caspases, and PARP cleavage [364]. All of these events were required for the LtxA-induced apoptotic and necrotic forms of cell death of Jurkat cells [364]. Experiments in a mouse model of pyelonephritis showed that animals continuously treated with a P2X_7_ receptor antagonist or lacking P2X_7_ receptors were protected against renal fibrosis after pyelonephritis with HlyA-producing *E. coli*, most likely due to reduced macrophage infiltration in the kidneys of P2X_7_-deficient mice [365].

In 2012, Dhakal et al. showed that sublytic concentrations of HlyA trigger rapid degradation of host cell proteins involved in cell–cell and cell–matrix interactions, inflammatory responses, and survival pathways [344]. The insertion of HlyA into the membrane of human bladder epithelial cells and macrophages stimulated rapid proteolysis of the cytoskeletal scaffolding protein paxillin, key components of the proinflammatory nuclear factor kappa B (NFκB) pathway, and a subset of other regulatory proteins. Proteolysis of these proteins required HlyA action-mediated activation of host serine proteases, of which the serine protease mesotrypsin was involved in the degradation of paxillin. Moreover, the HlyA intoxication of cells also stimulated the rapid activation of caspases involved in the execution of apoptosis. Thus, the HlyA-induced proteolysis of host proteins plays an important role in the functionality and survival of both epithelial cells and phagocytes [344].

Infections of colonic monolayers and native rat colon with extraintestinal pathogenic *E. coli* secreting HlyA showed that the bacteria could translocate through the cell layers 2 h after inoculation [348]. The sites of translocation were small defects in epithelial integrity, called focal leaks, which were not observed when the cell layers were infected with mutant strains lacking HlyA. Thus, the HlyA-induced focal leaks in the colonic epithelial barrier represented a novel route of bacterial translocation [348]. Later, the same group demonstrated that mice infected with HlyA-secreting *E. coli* showed an increase in focal leak areas in the colonized colons compared to the HlyA-deficient mutant, suggesting that HlyA impairs intestinal barrier function via induction of focal leaks in the epithelium in vivo [349]. Recently, the same group presented data that could explain the induction of focal leaks in the epithelial layer [366]. It was shown that the infection of the human colon carcinoma cell line Caco-2 with HlyA-secreting but not HlyA-deficient *E. coli* bacteria induced inhibition of PTEN (phosphatase and tensin homolog deleted on chromosome 10), a phosphatase that dephosphorylates membrane phosphatidylinositol-3,4,5-trisphosphate (PIP3) and plays a role in regulation of cell polarity. The inhibition of PTEN resulted in a decrease in membrane phosphatidylinositol-4,5-bisphosphate (PIP2) and induced changes in cell polarity. This resulted in the disorganization of barrier-forming junctional complexes and increased epithelial permeability, followed by increased epithelial cell detachment. Thus, these host cell processes dysregulated by HlyA could induce focal leaks in the intestinal epithelium, potentiating intestinal diseases caused by pathogenic *E. coli* strains [366].

Unlike other RTX toxins, the bifunctional CyaA toxin can subvert cell physiology very efficiently by translocation of its unique AC enzyme domain to the cytosol, where it catalyzes the unregulated conversion of ATP to a supraphysiological concentration of cAMP [122,123,199]. The signaling of cAMP then inhibits the bactericidal activities of immune cells such as complement-mediated phagocytosis, oxidative burst, or the formation of neutrophil extracellular traps and triggers apoptosis of macrophages and/or dedifferentiation of macrophages into monocyte-like cells [57,367,368,369,370,371,372,373,374]. The translocation of the AC domain of CyaA across the membrane also leads to the influx of extracellular calcium ions into the cytosol of monocytic cells [332]. The calcium influx then rescues CyaA from rapid endocytic removal of the toxin from the cytoplasmic membrane and enables protracted cell permeabilization by toxin pores [375]. The efflux of cellular potassium ions through the pores then further decreases the removal of the pores from the membrane, thereby further increasing cell permeabilization and potassium efflux in a positive feedback loop [375]. Using different CyaA variants, the pore-forming activity of the toxin was shown to contribute to its cAMP-elevating capacity to maximize the overall cytolytic capacity of CyaA in vitro [376,377]. Moreover, the pore-forming activity of CyaA induced the activation of the NALP3 inflammasome and the release of IL-1β by dendritic cells, most likely due to the efflux of cellular potassium ions through the toxin pores [342]. In addition, the pore-forming activity of the enzymatically inactive CyaA toxoid triggered maturation of dendritic cells that involved the activity of the mitogen activated protein kinases JNK (Jun N-terminal kinase) and p38 but was independent of Toll-like receptors and inflammasome signaling [346]. Interestingly, in the course of *B. pertussis* infections in vivo, the cAMP-elevating capacity of CyaA prevailed over the pore-forming activity of the toxin, which appeared to play an auxiliary role in the biological activity of CyaA [328,329].

Little is known about the molecular mechanisms by which RtxA modulates essential cellular functions or about the role of the toxin in the pathogenic process. Previous studies suggest that RtxA may play multiple roles in the pathogenesis of *K. kingae* disease, including upper respiratory tract colonization, bloodstream invasion, and target tissue damage [23,37,39]. In 2014, Chang et al. used *K. kingae* strain PYKK081 and its isogenic RtxA-deficient KKNB100 mutant to investigate the role of RtxA in *K. kingae* virulence following intraperitoneal injections in 7-day-postnatal rats [28]. Whereas the parental strain PYKK081 caused lethal disease with bacteremia, rapid weight loss, and abdominal necrotic lesion formation, the mutant strain KKNB100 was less toxic to rats and showed no signs of bacteremia, weight loss, or histopathological changes. In contrast to rats injected with PYKK081, animals injected with KKNB100 had significantly increased numbers of circulating leukocytes, suggesting that RtxA contributes to leukocyte depletion [28]. Several other studies have shown that RtxA is cytotoxic to various cultured cells (e.g., hypopharyngeal FaDu epithelial cells, laryngeal HlaC-78 squamous cells, synovial SW 982 cells, bone osteosarcoma U-2 OS epithelial cells, monocyte/macrophage RAW 264.7 cells, monocyte THP-1 cells, etc.), most likely due to the formation of cation-selective pores in the cell membrane [23,36,37,39]. However, the potential effects of RtxA action on major cellular components of signal transduction pathways, cell cycle, actin cytoskeleton, immune and inflammatory responses, barrier-forming junctional complexes, and others have not been investigated. Therefore, a detailed study of the RtxA toxin is urgently needed to improve our understanding of the molecular mechanisms responsible for the pathogenesis of *K. kingae* disease.

## 11. Conclusions

The Gram-negative bacterium *K. kingae* was recognized as part of the commensal oropharyngeal flora after its discovery in 1960 and was initially considered a rare cause of infection. However, advanced culture techniques and newly developed molecular detection methods revealed that *K. kingae* is a common cause of septic arthritis and osteomyelitis in children and can also cause other invasive diseases such as infective endocarditis, bacteremia, meningitis, ocular infections, pneumonia, pericarditis, or peritonitis. However, the increasing number of reports showing that *K. kingae* is an important cause of various pediatric diseases has shown how surprisingly little we know and understand about the pathophysiological mechanisms underlying the colonization and invasive capacities of *K. kingae*. In 2007, *K. kingae* was shown to be toxic to various cell types isolated from different mammalian species, and the cytotoxic effect was attributed to the cytotoxin RtxA. The *rtx* locus, which encodes RtxA and other proteins required for its posttranslational activation and secretion from the bacterial cell, was detected in all clinical isolates of *K. kingae*. Using the infant rat model and the RtxA-deficient mutant KKNB100, it was demonstrated that RtxA is a key virulence factor of *K. kingae*. RtxA belongs to a broad family of pore-forming RTX cytotoxins, which are secreted by many Gram-negative pathogens and share several functional domains and characteristic segments. These include the N-terminal pore-forming domain, the acylated segment, the typical C-terminal calcium-binding RTX domain, and the C-proximal secretion signal recognized by the T1SS. While the RTX toxins such as HlyA of *E. coli*, CyaA of *B. pertussis*, and LtxA of *A. actinomycetemcomitans* have been intensively characterized and established as key virulence factors over the past decades, RtxA is among the least studied members of the RTX toxin family, and little is known about its role in the pathogenic process. Recently, we showed that the binding of RtxA to cells depends on cell surface oligosaccharides and membrane cholesterol, but not on leukocyte-restricted β_2_ integrins, which are recognized as cell surface receptors by some other RTX cytotoxins. This explained the previously observed broad cellular specificity of RtxA and showed that the toxin belongs to the group of broadly cytolytic RTX hemolysins. After binding to cells, RtxA inserts into the cell membrane by an unexplored mechanism and forms cation-selective membrane pores that trigger a cation flux that disrupts normal cell physiology and eventually leads to cell death. However, how the cytotoxicity of RtxA contributes to the pathogenesis of *K. Kingae* disease is still poorly understood. Based on recent findings, we can hypothesize that RtxA may be involved in the process of colonization of the upper respiratory tract, disruption of the respiratory epithelial barrier to allow *K. kingae* to invade the bloodstream, paralysis of host innate immunity, and damage to target tissues once the bacterium is disseminated to distant sites of the body. Further detailed study of the RtxA toxin is therefore needed to improve our understanding of the molecular mechanisms involved in the pathogenesis of *K. kingae* infections.

## Figures and Tables

**Table 2:** *K. kingae* phasevarion.

*K. kingae* Genes	*modK1* ON ^1^	*modK1* OFF ^1^	Host Genes	*modK1* ON ^1^	*modK1* OFF ^1^
***rtxA***	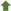	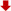	***IL-1β***	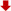	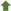
***groEL***	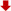	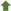	***TNF***	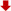	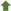
***dnaK***	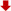	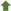	***IL-8***	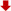	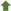

^1^ Green arrows, upregulation; red arrows, downregulation.
